# Long Term Weight Loss Diets and Obesity Indices: Results of a Network Meta-Analysis

**DOI:** 10.3389/fnut.2022.821096

**Published:** 2022-04-05

**Authors:** Jana Jabbour, Yasmin Rihawi, Assem M. Khamis, Layal Ghamlouche, Bayan Tabban, Gloria Safadi, Nour Hammad, Ruba Hadla, Marwa Zeidan, Dana Andari, Riwa Nour Azar, Nadine Nasser, Marlene Chakhtoura

**Affiliations:** ^1^Nutrition Department, School of Health Sciences, Modern University for Business and Sciences, Beirut, Lebanon; ^2^Department of Clinical Nutrition, American University of Beirut Medical Center, Beirut, Lebanon; ^3^York Medical School, University of Hull, York, United Kingdom; ^4^Research & Programmes Department, Qualisus Consulting, Byblos, Lebanon; ^5^Center for Research on Population and Health, Faculty of Health Sciences, American University of Beirut, Beirut, Lebanon; ^6^Duke Global Health Institute, Duke University, Durham, NC, United States; ^7^Department of Health Management and Policy, Faculty of Health Sciences, American University of Beirut, Beirut, Lebanon; ^8^Faculty of Medicine, American University of Beirut, Beirut, Lebanon; ^9^Department of Nutrition and Dietetics, Faculty of Health Sciences, Beirut Arab University, Beirut, Lebanon; ^10^The European School of Management and Technology, Berlin, Germany; ^11^Access to Nutrition Initiative, Utrecht, Netherlands; ^12^Calcium Metabolism and Osteoporosis Program, WHO Collaborating Center for Metabolic Bone Disorders, American University of Beirut Medical Center, Beirut, Lebanon

**Keywords:** diet, obesity, Acceptable Macronutrient Distribution Ranges (AMDR), weight loss, waist circumference, body mass index (BMI)

## Abstract

**Background:**

Scientists have been investigating efficient interventions to prevent and manage obesity. This network meta-analysis (NMA) compared the effect of different diets [moderate macronutrients (MMs), low fat/high carbohydrate (LFHC), high fat/low carbohydrate (HFLC), and usual diet (UD)] on weight, body mass index (BMI), and waist circumference (WC) changes at ≥12 months.

**Methods:**

We searched Medline, Embase, PubMed databases, and the Cochrane Library. We systematically assessed randomized controlled trials (RCTs) evaluating dietary interventions on adults (mean BMI ≥ 25 kg/m^2^) receiving active dietary counseling for ≥12 months. We pooled the data using a random-effect NMA. We assessed the quality of the included RCTs using the Cochrane risk of bias (ROB) tool.

**Results:**

We included 36 trials, 14 of which compared HFLC with MM diets. Compared with UD, all diets were associated with a significant weight loss (WL) at ≥12 months, HFLC [mean difference in kg (95% *CI*): −5.5 (−7.6; −3.4)], LFHC [−5.0 (−7.1; −2.9)] and MM [−4.7 (−6.8; −2.7)]. HFLC, compared with MM diet, was associated with a slightly higher WL (of −0.77 kg) and drop in BMI (of −0.36 kg/m^2^), while no significant difference was detected in other dietary comparisons. WC was lower with all diets compared to UD, with no significant difference across specific diets. There was no significant interaction of the results with the pre-specified sub-groups. The ROB was moderate to high, mostly related to unclear allocation concealment, high dropout rate and unclear or lack of blinding of participants, providers, and outcome assessors.

**Conclusion:**

Dietary interventions extending over ≥12 months are superior to UD in inducing weight, BMI and WC loss. HFLC might be associated with a slightly higher WL compared with MM diets.

**Systematic Trial Registration:**

https://www.crd.york.ac.uk/prospero/display_record.php?RecordID=103116, PROSPERO (CRD42018103116).

## Introduction

Obesity has almost increased three times in the last three decades to reach pandemic levels (1). Obesity is associated with a decreased lifetime expectancy of 5–20 years, depending on the severity and the presence of comorbidities (2–4). With more than half of the world population being overweight or obese, scientists are continuously exploring efficient interventions to prevent and manage this pandemic (5, 6).

Diet therapy remains one of the cornerstones of the multi-disciplinary approach to weight management. However, obesity treatment guidelines have variable recommendations regarding the most appropriate diet (Supplementary Table S1). While almost all agree on a reduced calorie meal plan, and a modification of macronutrient composition to enhance the dietary adherence and improve the metabolic profile, (7) (Supplementary Table S1), there is still no consensus yet on the most optimal macronutrient dietary pattern for weight management.

A network meta-analysis (NMA) is a meta-analysis (MA) technique that allows evaluation of at least three interventions in one analysis, using both direct and indirect comparisons (8). It is an “evidence synthesis method” gaining interest in the field of nutrition research (9). Two recent NMAs of randomized controlled trials (RCTs) assessed the short term (6–12 months) effect of different diets (10, 11). The first NMA included the following diet categories: lifestyle, exercise, attitudes, relationships, nutrition (LEARN), low carbohydrate, low fat, and moderate macronutrients (MMs) (10). The results (*n* = 7,286 participants) revealed that, compared with no diet, and as expected, any dietary intervention resulted in a significant weight loss (WL) at 6 months, of 5.1–8.7 kg (10). The WL response was attenuated at 12 months, with a weight reduction of 1–2 kg less compared with the 6-month follow up (10). The comparison of diets with different macronutrient composition between each other showed that a low carbohydrate diet was better than a MMs diet, at 6 and 12 months, with a small difference in mean WL of 1.9 and 1.5 kg, respectively, while none of the other comparisons reached significance (10). The more recent NMA (*n* = 21,942 participants) by Ge et al. included trials published until September 2018 and similarly showed that the low carbohydrate, low fat, and MM diets were superior to a usual diet (UD) at 6 months (WL of 4.6, 4.4, and 3.1 kg, respectively), with no significant difference comparing diets among each other's (11). WL decreased by 1.5 kg on average at 12 months compared with the 6-month assessment point (11). Several systematic reviews and meta-analyses (SR/MA) assessed the long term weight reducing effects of various diets beyond 12 months follow up (12–17). Two SR/MAs compared a low fat diet to any higher fat diet, including UD, and did not demonstrate any significant difference in the achieved weight at follow-up (15, 18). Two other SR/MAs compared a high protein/low or very low carbohydrate diet to other diets and showed a significant MD in WL of 0.4–0.9 kg, favoring the former diet (12, 14). One SR/MA compared Atkins, Weight Watchers diet, South Beach and Zone, and demonstrated a modest and comparable WL across all (13). The main limitations of the aforementioned SR/MAs assessing the long-term effects of dietary interventions stem from the inclusion of RCTs on patients with chronic diseases, such as diabetes mellitus (DM) and cancer, that might affect the WL response, the inclusion of RCTs with an active diet intervention extending over <1 year, or the lack of a systematic description and investigation of the effect of co-interventions, such as exercise and behavioral therapy.

Given the heavy burden of obesity, its chronic relapsing nature (19), and the lack of consensus on the most optimal diet composition, if any, for weight reduction, this SR/NMA aims at evaluating the association of long term dietary interventions, categorized using the Acceptable Macronutrient Distribution Ranges (AMDR), with changes in weight parameters. The AMDR, recommended by the Institute of Medicine (IOM), is widely used by clinicians and defines the ranges of macronutrient contribution to energy intake that have been linked to a lower risk of chronic diseases (20, 21).

## Methods

The protocol for this SR/NMA followed the preferred reporting items for systematic reviews and meta-analyses (PRISMA), and was registered on PROSPERO (CRD42018103116) (22).

### Eligibility Criteria

This section describes the Population, Intervention, Control, and Outcome (PICO) elements and details the eligibility criteria of this SR. We selected RCTs as we expected to have a complete summary of the evidence on the topic by gathering data from interventional studies. We included RCTs conducted in adults with overweight/obesity (mean body mass index (BMI) at baseline ≥ 25 kg/m^2^) *(population)*, comparing an active dietary intervention of ≥12(±1) months *(intervention)*, to another dietary regimen or UD *(control)*, and reporting on one or more outcomes of interest, change in weight, BMI, or waist circumference (WC), at ≥12 months follow-up *(outcome)*. We included only papers written in English. To assess the effect of dietary interventions in healthy individuals, we excluded trials where the majority of participants (>75%) were pregnant women, had chronic diseases (such as cancer, diabetes mellitus, advanced liver, or renal disease), or received medications inducing weight gain (such as anti-psychotic drugs), as these conditions are expected to affect the WL response. We excluded trials if the intensity of the intervention or the co-intervention differed between arms or in case a detailed description of the intervention (such as duration and macronutrient composition) was not provided in the trial or trial protocol publication. In addition, we excluded interventions consisting of a change in a single food item or that solely relied on the supplementation, as we aimed to assess the impact of comprehensive dietary changes. Exclusion criteria included RCTs assigning very-low-calorie diets (<800 Kcal/day) or meal replacement liquids (since such diets are not recommended as long term interventions), and RCTs where diets were assigned based on participants' genetic profiles (22).

### Search Strategy

We conducted a systematic search in the following electronic databases Medline, Embase, PubMed, and the Cochrane Library, without time restriction and until December 2020. We used Medical Subject Heading (MeSH) terms and keywords relevant to the dietary intervention and overweight or obesity (as shown in Supplementary Text S1). Moreover, we manually searched the references of SRs on the topic to identify any potentially relevant studies that may have been missed. We contacted experts in the field and searched ClinicalTrials.gov for potentially completed and non-published trials.

### Data Screening and Abstraction

We conducted the screening of citations and full texts, and data abstraction in duplicate and independently, using standardized forms, prepared a priori. At each step, we conducted a calibration exercise until discrepancy rate between reviewers got to <5%. The calibration exercise involved training and cross training of the researchers on the eligibility criteria and data abstraction, to make sure that the process was standardized. We prepared screening sheets for each step based on our research question. At all stages, we resolved disagreement between reviewers by discussion and through intervention from content experts (MC and JJ).

### Risk of Bias (ROB) and Publication Bias Assessment in the Included RCTs

We assessed the risk of bias (ROB) of the included RCTs in duplicate and independently using the Cochrane ROB assessment tool (23). We assessed the risk of publication bias by visually checking the symmetry of the funnel plot of the included studies in the traditional meta-analyses for comparisons including ≥10 RCTs. In the funnel plot, for each trial, we plotted the effect by the inverse of its SE.

### Statistical Considerations and Analyses

We presented the characteristics of the included RCTs as counts (percentages) and means (ranges or SD) for categorical and continuous variables, respectively. We used complete case analysis in the quantitative analysis. We categorized the dietary interventions of the included RCTs using the AMDR as defined by the IOM (20). MM diets referred to diets where all macronutrients were within the AMDR ranges: carbohydrate (45–65% of energy), protein (10–35% of energy), and fat (20–35% of energy). High fat/low carbohydrate (HFLC) diets had a total fat percentage that exceeded the AMDR range (>35% of energy) and/or carbohydrate percentage below the lower AMDR limit (≤45% of energy). Low fat/high carbohydrate (LFHC) diets had a total fat percentage below the lower AMDR limit (≤20% of energy) and/or carbohydrates percentage exceeding the upper AMDR limit (>65% of energy). UD were control diets where participants were asked not to change their dietary intake from their usual lifestyle.

We used Bayesian random-effects model to assess the pooled direct and the NMA estimates. We derived the latter using Markov chain Monte Carlo simulation techniques (24, 25). The outcome measures of interest were the change in weight (kg), the change in BMI (kg/m^2^), and the change in WC (cm). When the change in the outcome measure was not reported, we used baseline and study end mean (SD) values of the outcome measure to calculate the mean difference (MD) of the change of this outcome. When the mean of the change was available but the SD was missing, we calculated the SD using the SE, 95% CI and/or *p*, when available, as suggested by the Cochrane Handbook (26). For the diets included in the NMA, we assessed the likelihood for every diet to be ranked first, second, etc. (27). We evaluated the statistical heterogeneity between studies for direct comparisons using *I*^2^ statistic, defining moderate heterogeneity for *I*^2^ of 40–70% and high for *I*^2^ > 70%.

For the traditional MA, we calculated the MD and 95% CI of continuous variables, when at least 2 RCTs were included in a given comparison, using a random-effects model. We used imputation methods when needed, as described above. We explored the reasons for moderate to high heterogeneity, by conducting sub-group analyses for the following variables, by outcome and by comparison, as applicable, when a given sub-group included at least 2 RCTs: gender (>75% of participants being men or women), baseline mean BMI (<30 vs. ≥ 30 kg/m^2^), age category (younger vs. older adults based on a cutoff point of 50 years, as a surrogate of menopausal status), intervention duration (12 months vs. 13–24 months), and the presence vs. the absence of concomitant exercise and/or behavioral prescription. We considered any explicit instruction on exercise, whether advised or supervised, as a physical activity (PA) co-intervention. Similarly, we considered any kind of behavioral support received, irrespective of the provider, as a behavioral co-intervention. We did not have data to explore the impact of compliance, dietary fiber content, and dietary restriction vs. *ad libitum* on the outcomes of interest. All the sub-group analyses were pre-specified in the protocol (22), with the exception of gender.

We used RStudio v1.4.1106 (Integrated Development for R. RStudio, PBC, Boston, MA) for the NMA and the league tables. We used Stata 17 to generate the network nodes (StataCorp. 2021. Stata Statistical Software: Release 17. College Station, TX: StataCorp LLC). We conducted the traditional meta-analysis, subgroup analyses, and funnel plots on Review Manager [(RevMan) (Computer program) Version 5.3. Copenhagen: The Nordic Cochrane Center, The Cochrane Collaboration, 2014].

## Results

**Figure 1:**
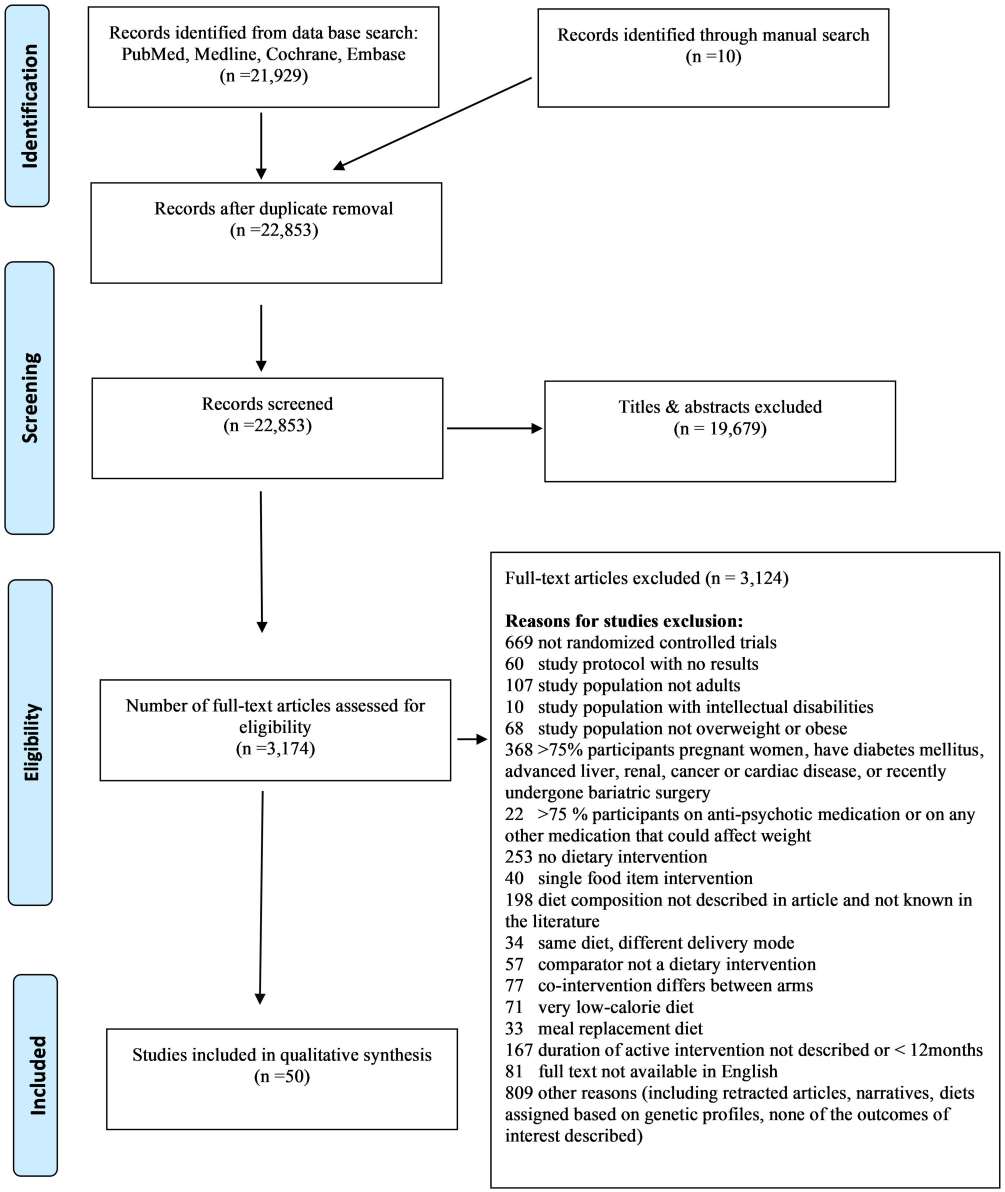
Flow diagram of included studies.

The search strategy yielded 22,929 citations. After removal of duplicates, we screened 22,853 citations, 3,082 full text, and included 50 publications relevant to 36 trials (Figure 1). We identified 24 RCTs reporting on weight change (total *n* = 4,916 participants), 17 RCTs on BMI change (total *n* = 3,260 participants), and 15 RCTs on WC change (total *n* = 2,734 participants), and comparing diets with different macronutrient distribution between each other or to UD, at ≥12 months follow-up.

### Characteristics of the Included RCTs

Supplementary Table S2 showcases the detailed study characteristics of each of the included RCTs. Table 1 presents a summary of the characteristics of the included RCTs. The comparison of MM to HFLC diets included the largest number of RCTs. The sample size of the RCTs ranged between 7 and 318 participants per arm and the duration of the active intervention spanned over 12–24 months. The range of mean age of participants was 22–67 years, and the range of mean BMI at baseline was 25.9–43.6 Kg/m^2^. The majority of RCTs included both genders, and women represented >50% of the population, with the exception of 3 RCTs (28–30). Most of the trials were conducted in North America (44%) and Europe (28%). Furthermore, seventeen RCTs included participants with cardio-metabolic co-morbidities (e.g., coronary heart disease, hypertension, dyslipidemia, and metabolic syndrome) in <40% of the population. There were few exceptions where the majority of participants had metabolic syndrome (65–100% of the population) (31–35), hypertension (81% of the population) (33), and hyperinsulinemia (100% of the population) (36). The delivery of the dietary intervention was achieved in most of the trials through face-to-face individual and/or group assessment and education. Few trials incorporated a follow-up over the phone and/or *via* email (29, 37–41). Behavioral therapy and PA were co-interventions in 39 and 36% of the RCTs, respectively, and 8 RCTs had both co-interventions administered concomitantly (Table 1). Nine RCTs administered diets that have a similar macronutrient composition falling within the same AMDR classification, and therefore were not included in our quantitative analysis (35, 36, 41–47).

**Table 1:** Summary of the population and intervention characteristics of the included randomized controlled trials (RCTs).

**Variable**	**Results**
	**Range of means**
Age (years)	22–68
BMI (Kg/m^2^)	26–44
	***N*** (%)^*^
Gender	
Women only	7 (19)
Men only	4 (11)
Both	25 (69)
Continent	
Australia &New Zealand	7 (19)
Asia	3 (8)
Europe	10 (28)
North America	16 (44)
Diet categories comparisons	
MM vs. Usual diet	4 (11)
MM vs. LFHC	3 (8)
MM vs. HFLC	14 (39)
MM vs. MM	9 (25)
HFLC vs. HFLC	1 (3)
HFLC vs. LFHC	2 (5.5)
MM vs. LFHC vs. HFLC	2 (5.5)
LFHC vs. Usual diet	1 (3)
Study duration	
12 months	28 (78)
13–24 months	8 (22)
Mode of education¥	
Individual, face to face	19 (53)
Group, face to face	19 (53)
Mobile/email	6 (17)
Others	7 (19)
Behavioral co-intervention	14 (39)
Physical activity co-intervention	12 (33)

*BMI, Body Mass Index; MM, Moderate Macronutrient; HFLC, High Fat Low Carbohydrate; LFHC, Low Fat High Carbohydrate*.

* *Percentages calculated out of the 36 included trials*.

¥ *Percentages add up to more than 100% as there are trials that included several modes of education*.

The compliance to the dietary intervention was assessed in about 50% of the trials using a variety of methods, the most common being food records (32, 40, 43, 48–50), followed by food frequency questionnaires and dietary recalls (32, 35, 51), urine urea nitrogen (41, 52, 53), and educational sessions' attendance (30, 32, 33, 54). The dropout rate was reported in most studies. It was <20% in 20 RCTs, and larger reaching 30–60% in 14 RCTs. The highest dropout rates were observed in one RCT with young female participants (18–25 years) (55) and other trials with participants following HFLC diets (41, 49, 55, 56). When the funding was described, the source consisted of national or international scientific organizations, with the exception of one trial funded by a private company (55).

Supplementary Table S3 features the ROB assessment of the included RCTs. Most studies (72%) had a “High” overall ROB. The high ROB was related to a poor description or lack of appropriate allocation concealment and blinding of participants/personnel and outcome assessors', high dropout rate, and incomplete outcome reporting. Seventeen RCTs have their protocols posted online. Among the other risks of bias, the imbalance in baseline characteristics was the most common limitation. We were able to assess the risk of publication bias only for the comparison of HFLC to MM, as it included more than 10 RCTs (Supplementary Figures S1–S3). The funnel plots revealed asymmetry in the publications reporting weight changes. No asymmetry was noted for RCTs describing BMI and WC changes.

### Changes in Weight, BMI, and WC

**Figure 2:**
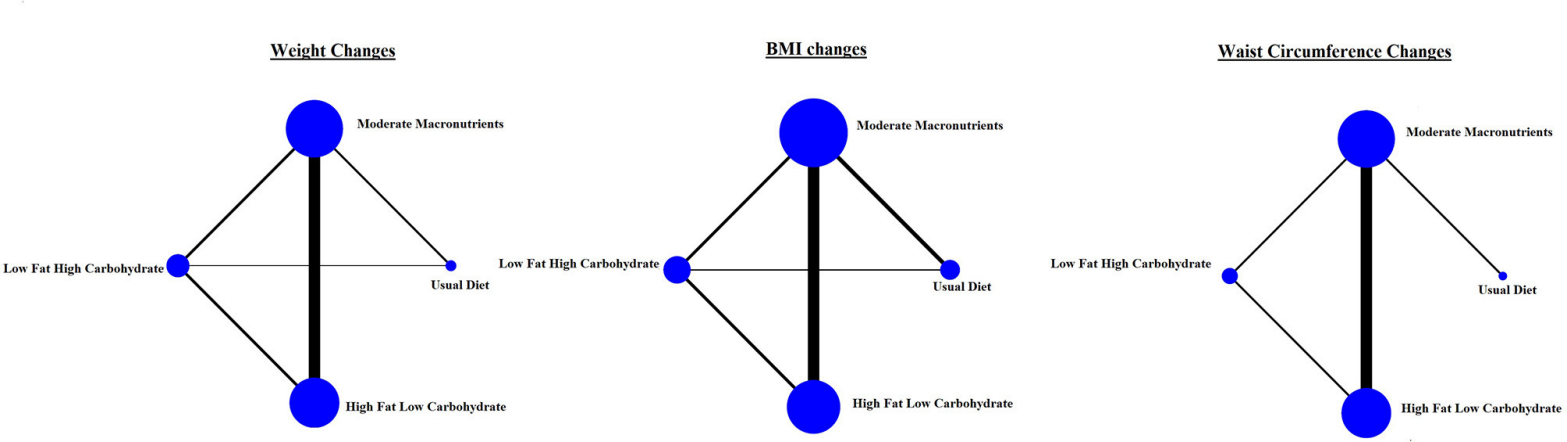
Network plot of included studies.

We conducted an NMA for each of the outcomes of interest (Figure 2). The largest number of direct comparisons was between MM and HFLC diets for all outcome measures (Figure 2). All estimates were fed by direct and indirect comparisons, with the exception of the one derived from UD vs. HFLC diet for the weight, BMI, and WC changes, and the one derived from UD vs. LFHC diet for the WC change, that were only based on indirect comparisons.

We identified 24 RCTs reporting on the weight changes (total *n* = 4,916 participants) at ≥ 12 months of follow-up (28, 29, 31–34, 37–40, 49–51, 53–63). The NMA revealed that, compared with UD, all diets were associated with a significant and comparable WL, at 12 months and beyond (Table 2), HFLC diets [MD (95% *CI*): −5.5 (−7.6; −3.4)] kg, LFHC diets [−5.0 (−7.1; −2.9)] kg and the MM diets [−4.7 (−6.8; −2.7)] kg (Table 2). When comparing dietary interventions between each other, the only significant difference was for the HFLC diets, associated with a higher WL compared with MM diets [MD (95% *CI*): −0.77 (−1.3; −0.19)] kg. The HFLC diet had the highest probability of being superior to all other diets (82%). The ROB of the included studies was moderate to high, mostly related to unclear allocation concealment and blinding, and high dropout rate of 20–60% (Supplementary Figure S1).

**Table 2:** League table of network meta-analysis results of weight changes (Kg) and BMI changes (Kg/m^2^) at study end (≥ 12 months).

			**BMI (Kg/m^**2**^)**	
**Weight (Kg)**	**Usual diet** (W 0.09; BMI 0)^*^	−2.1 (−2.8:−1.4)	−1.8 (−2.5: −1.0)	−1.7 (−2.4:−1.1)
	−5.5 (−7.6: −3.4)	**High fat low carbohydrate diet (W 0.82; BMI 0.96)** ^*^	0.30 (−0.18: 0.79)	0.36 (0.05: 0.67)
	−5.0 (−7.1:−2.9)	0.47 (−0.32:1.3)	**Low fat high carbohydrate diet** (W 0.80; BMI 0.58)^*^	0.06 (−0.42:0.54)
	−4.7 (−6.8:−2.7)	0.77 (0.19:1.3)	0.77 (−0.38:1.9)	**Moderate macronutrient** diet (W 0.29; BMI 0.47)^*^

*BMI, body mass index; W, weight*.

*Values corresponding to weight changes are below or to the left of the diet categories. Values corresponding to BMI changes are above and to the right of the diet categories*.

* *Numbers between parentheses refer to the probability of the diet being selected the best*.

Moreover, 17 RCTs presented BMI changes (total *n* = 3,260 participants) at ≥ 12 months (29, 31, 34, 37–39, 49, 50, 54, 57–59, 61, 62, 64–66). The NMA on BMI change showed results similar to WL. There was a significant drop in BMI across all diets compared with UD, HFLC [−2.1 (−2.8; −1.4)] kg/m^2^, LFHC [−1.8 (−2.5; −1.0)] kg/m^2^, and the MM diets [−1.7 (−2.4; −1.1)] kg/m^2^ (Table 2). When comparing dietary interventions between each other, the only significant difference was for the HFLC diets, associated with a significantly lower BMI compared with MM diets [MD (95% *CI*): −0.36 (−0.67; −0.05) kg/m^2^]. The HFLC diets had the highest probability of being superior to all other diets (96%). The ROB of the included studies was moderate to high, mostly related to unclear sequence generation, allocation concealment and blinding, and incomplete data reporting, with a high dropout rate of 20–60% and other bias (Supplementary Figure S2).

We identified 15 RCTs reporting on WC change (total *n* = 2,734 participants) at ≥12 months (29, 31, 34, 37, 38, 49–51, 53–55, 57–59, 65). The results on WC changes echoed those of weight and BMI changes, with the HFLC associated with the highest changes [MD (95% *CI*): −5.6 cm (−8.6; −2.6)], followed by LFHC and MM diets, compared with UD. Yet, there was no significant difference in WC when diets were compared with each other (Table 3). The ROB of the included studies was moderate to high mostly related to unclear allocation concealment and blinding, and high dropout (Supplementary Figure S3).

**Table 3:** League table of network meta-analysis results of waist circumference changes (cm) at study end (≥ 12 months).

Waist circumference(cm)	Usual diet (0)^*^			
	−5.6 (−8.6:−2.6)	High fat low carbohydrate diet (0.95)^*^		
	−5.2 (−8.4:−2.1)	0.36 (−0.85:1.6)	Low fat high carbohydrate diet (0.58)^*^	
	−5.0 (−7.9:−2.1)	0.61 (−0.14:1.4)	0.25 (−0.97:1.5)	Moderate macronutrient diet (0.47)^*^

* *Numbers between parentheses refer to the probability of the diet being selected the best*.

### Sub-group Analyses

We conducted subgroup analyses for the MM vs. LFHC comparison, as it had a moderate to high heterogeneity in the traditional MA for weight change (*I*^2^ 56%), BMI change (*I*^2^ 77%), and WC change (*I*^2^ 40%) (Supplementary Table S4). There was a larger drop in BMI with a longer intervention duration of 12 months or more [MD −0.73 (−1.23; −0.27) kg/m^2^], compared with a duration of 12 months only [MD −0.19 (−0.36; −0.02) kg/m^2^], of borderline significance with a *p*-subgroup analysis 0.05. However, this finding was not reproduced for other outcome measures. Sub-group analysis by the type of co-intervention showed a trend for a larger difference in the mean change in WC in trials that administered both co-interventions (PA and behavioral therapy), *p*-subgroup analysis 0.05. We did not find any interaction by gender. We detected a consistent trend for a slightly larger effect in younger (age <50 years), compared with older (age ≥50 years) individuals, across various outcome measures. We could not assess the impact of baseline BMI given the narrow range of mean BMI (of 30–40 kg/m^2^) in most studies.

### Adverse Events

The adverse events were reported only in 6/36 trials (28, 33, 35, 52, 53, 57). Most adverse events were not related to the dietary interventions. Some diet related adverse events included increased urine micro-albumin to creatinine ratio in patients following high-protein diets (52) and hypoglycemia following an oral glucose tolerance test (57).

## Discussion

This NMA exclusively analyzes the long-term (≥ 12 months) effect of dietary interventions with active counseling, using the IOM macronutrient categorization brackets for diets categorization. The conduct of such an NMA is important given the wide utilization and reference of the AMDR (20). All diet categories (MM, LFHC, and HFLC), compared with UD, were associated with a significant WL of about 5 kg, a significant drop in BMI of 2 kg/m^2^, and a significant drop in WC of 5 cm, at 12–24 months follow-up. Diets did not differ among each other, with the exception of the HFLC diet that was slightly better than MM diet, with a larger WL (of 0.8 kg) and BMI loss (0.4 kg/m^2^). Since these differences in weight and BMI have a minimal clinical significance, our findings confirm that all diets have the same efficacy on weight management, and provide the evidence for obesity guidelines recommendations, not favoring a specific diet beyond the one that would optimize patient adherence and the adoption of healthy eating patterns (7, 67). Our conclusion contrasts with what diet advertisements claim to the public about the superiority of certain diets over others (68–70). Popular diets may be helpful as a jumpstart but do not affect long-term weight and WC changes (71).

Results of this NMA are aligned with those of two previous NMAs that assessed the effect of dietary interventions at 6–12 months follow-up (10, 11). Both NMAs showed that the different diet categories were superior to UD at 12 months, with a drop in weight (of 5–7 kg) and BMI of around 2 kg/m^2^, similar to our findings. When comparing diets among each other, both NMAs showed a slightly higher effect of a low carbohydrate diet compared with the MM diet (10, 11), as demonstrated in our findings. Ge et al. showed a higher effect of a low-fat diet compared with an MM diet (11). While our NMA assessed the effect of dietary interventions of at least 12 months, in patients without chronic diseases potentially affecting WL, it confirmed the result of previous SR/MA on the topic, and hence showed comparable results, suggesting weight maintenance at a certain plateau with continuous dietary efforts beyond 12 months (72).

Our significant findings in one comparison only may be related to a higher power to detect significance given the larger number of included trials comparing HFLC to MM diets. The potential superiority of HFLC over an MM on weight and BMI changes may be explained by several reasons. A low-carbohydrate diet implies a higher protein intake and a reduced sugar consumption, and therefore more satiety (73). A HFLC diet may be associated with an higher secretion of the anorexigenic peptide YY hormone (74, 75), and a higher energy expenditure, compared with other diets (76). Furthermore, the carbohydrate-insulin model of obesity favors a low carbohydrate and a low glycemic index diet, allowing less fat deposition and higher energy expenditure, compared to a traditional low fat diet (77). However, this model has been criticized for being “too simplistic” (78), and further research is needed to explore the implication of this model in specific patient populations.

Aging is associated with a decrease in the total energy expenditure, and a change in various hormones that affect body composition and appetite (79). However, we did not detect a significant interaction by age, but a trend for a larger effect in younger individuals. Moreover, our subgroup analysis revealed a trend for better results in WC among recipients of PA and behavioral therapy interventions, compared with either one. Such findings highlight the importance of a multidisciplinary approach in weight management, such as diet, exercise, and psychologic support (80). Noteworthy, there was a wide heterogeneity in the intensity of delivery of the physical activity, consisting of education about healthy habits or supervised exercise sessions (29, 30, 34, 37, 44, 49, 51, 54, 55, 57, 59, 62). Similarly, the behavioral support consisted of counseling by behavioral therapists as well as support sessions provided by healthcare providers non-specialized in the behavioral field (34, 37, 39, 43, 44, 46, 50, 51, 53, 57, 59–62).

### Strengths/Limitations

To our knowledge, this is the most comprehensive review on the long-term effect (≥ 12 months) of diets, assessing the WL response, in the general population with overweight/obesity. We have used a rigorous approach in the identification, data abstraction, and analysis of relevant RCTs. Our findings are based on RCTs mostly derived from Western populations and therefore might not be generalizable to non-Western countries. In addition, with the exception of few trials conducted exclusively in men, women constituted the majority of the participants, and therefore, we were not able to explore the gender effect on the response to dietary interventions. Our limitations stem from challenges identified in the included RCTs, most of which were of low quality, with a high ROB. We could not assess the impact of the quality of RCTs on the results given the paucity of high-quality trials. Although the International Committee of Medical Journal Editors (ICMJE) required, as of 2005, trials protocol registration for publication, and same did the World Health Organization as of 2006, we identified several studies published after 2007, without an available published protocol. While blinding is an essential component in RCTs to reduce the risk of performance and detection bias, blinding of participants and dieticians to dietary interventions is very difficult, as previously recognized (81–83). There was a wide heterogeneity in the intensity and delivery mode of the dietary interventions, and this could have affected the WL response. Furthermore, compliance was assessed qualitatively in the majority of studies, implying the lack of accurate assessment of participants' adherence to dietary intervention. Few trials used quantitative methods and these included 24-h recall, self-reported food records, and food frequency questionnaires, and fewer ones chose urine urea nitrogen, urinary ketone levels, and respiratory quotient; the latter are preferred methods as they are subjected to less recall and social desirability biases. Due to the difference in the assessment methodology, there were no means of evaluating the relationship between adherence rate and changes in weight parameters. Finally, we noted an under-reporting of adverse events, as described previously (10, 11). Although various diet therapies are safe, limited data are available on their long-term effects (84).

## Conclusion

Compared with the usual diet, all dietary interventions allow a sustained modest WL during the follow-up of 12 months and beyond. A HFLC diet seems to be slightly better than a MM diet, while all other comparisons between diets yield similar results. A major limitation of the findings stems from the lack of compliance/adherence data, the wide variability of the delivery of dietary interventions, and the low quality of RCTs. A formal and standardized delivery of diet therapy, a qualitative assessment of participants' adherence to diets, efforts to improve on blinding of participants and researchers, and to reduce the participants' attrition are essential in future trials. While our findings apply to the general population of patients with overweight/obesity, the long-term impact of dietary approaches on patients with chronic diseases is worth investigation in a separate systematic review of the literature.

## Data Availability Statement

The raw data supporting the conclusions of this article will be made available by the authors, without undue reservation.

## Author Contributions

MC and JJ conceived, designed the study, coordinated data screening and abstraction, and wrote and reviewed the manuscript. MC, JJ, and AK analyzed and interpreted the data. DA, RA, RH, LG, NH, NN, GS, BT, YR, and MZ performed data screening. DA, RH, LG, NH, GS, BT, YR, and MZ performed data abstraction. YR and NH managed the data. All authors revised the article critically for important intellectual content, gave final approval of the version to be published, and agreed to be accountable for all aspects of the work. All authors contributed to the article and approved the submitted version.

## Conflict of Interest

LG is employed by Qualisus Consulting. The remaining authors declare that the research was conducted in the absence of any commercial or financial relationships that could be construed as a potential conflict of interest.

## Publisher's Note

All claims expressed in this article are solely those of the authors and do not necessarily represent those of their affiliated organizations, or those of the publisher, the editors and the reviewers. Any product that may be evaluated in this article, or claim that may be made by its manufacturer, is not guaranteed or endorsed by the publisher.
