# Supplementary material for: Long Term Weight Loss Diets and Obesity Indices: Results of a Network Meta-Analysis
Source: Front Nutr. 2022 Apr 5;9:821096. doi: 10.3389/fnut.2022.821096 (PMC9037142; doi:10.3389/fnut.2022.821096)
Supplement: Supplementary file 1 [file Data_Sheet_1.docx]

Supplementary Material

1. **Supplementary text**

**Text S1: Search Strategy**

The search was conducted in June 2018, and updated in December 2019 and December 2020. Below is a summary of the search strategy conducted on the below databases:

1) Pubmed

2) Medline

3) Cochrane

4) Embase

**PubMed:**

((obesity[mesh] OR obes*[tw] OR overweight[mesh] OR overweight[tw]) AND (diet[mesh:noexp] OR carbohydrate diet[tw] OR carbohydrate diets[tw] OR diabetic diet[tw] OR diabetic diets[tw] OR atherogenic diet[tw] OR atherogenic diets[tw] OR fat diet[tw] OR fat diets[tw] OR Mediterranean diet[tw] OR Mediterranean diets[tw] OR protein diet[tw] OR protein diets[tw] OR reducing diet[tw] OR reducing diets[tw] OR vegetarian diet[tw] OR vegetarian diets[tw] OR vegan diet[tw] OR vegan diets[tw] OR macrobiotic diet[tw] OR macrobiotic diets[tw] OR western diet[tw] OR western diets[tw] OR healthy diet[tw] OR healthy diets[tw] OR ketogenic diet[tw] OR ketogenic diets[tw] OR paleolithic diet[tw] OR paleolithic diets[tw] OR energy intake[tw] OR energies intake[tw] OR energetic intake[tw] OR portion size[tw] OR portions size[tw] OR portion sizes[tw] OR serving size[tw] OR serving sizes[tw] OR calorie restrict*[tw] OR caloric restrict*[tw] OR calories restrict*[tw]) AND (randomized controlled trial[pt] OR controlled clinical trial[pt] OR randomized[tiab] OR placebo[tiab] OR clinical trials as topic[mesh:noexp] OR randomly[tiab] OR trial[ti]) NOT (animals [mh] NOT (humans [mh] AND animals[mh])))

**Medline:**

Database: Ovid MEDLINE(R) Epub Ahead of Print, In-Process & Other Non-Indexed Citations, Ovid MEDLINE(R) Daily and Ovid MEDLINE(R)

Search Strategy: --------------------------------------------------------------------------------

1 exp Obesity/

2 obes*.tw,kw.

3 exp Overweight/

4 overweight*.tw, kw.

5 ((randomized controlled trial or controlled clinical trial).pt. or randomized.ab. or placebo.ab. or clinical trials as topic.sh. or randomly.ab. or trial.ti.) not (exp animals/ not humans.sh.)

6 diet/ or exp diet, carbohydrate loading/ or exp diet, diabetic/ or exp diet, atherogenic/ or exp diet, carbohydrate-restricted/ or exp diet, fat-restricted/ or exp diet, gluten-free/ or exp diet, high-fat/ or exp diet, mediterranean/ or exp diet, paleolithic/ or exp diet, protein-restricted/ or exp diet, reducing/ or exp diet, vegetarian/ or exp diet, macrobiotic/ or exp diet, vegan/ or exp diet, western/ or exp energy intake/ or exp caloric restriction/ or exp healthy diet/ or exp ketogenic diet/ or exp portion size/ or exp serving size/

7 (Diet? adj2 (carbohydrate or diabetic or atherogenic or fat or Mediterranean or protein or reducing or vegetarian or vegan or macrobiotic or western or healthy or ketogenic or paleolithic)).tw,kw. [mp=title, abstract, original title, name of substance word, subject heading word, floating sub-heading word, keyword heading word, protocol supplementary concept word, rare disease supplementary concept word, unique identifier, synonyms]

8 (energ* adj2 intake).tw,kw. [mp=title, abstract, original title, name of substance word, subject heading word, floating sub-heading word, keyword heading word, protocol supplementary concept word, rare disease supplementary concept word, unique identifier, synonyms]

9 ((Portion or serving?) adj2 size).tw,kw. [mp=title, abstract, original title, name of substance word, subject heading word, floating sub-heading word, keyword heading word, protocol supplementary concept word, rare disease supplementary concept word, unique identifier, synonyms]

10 (calori* adj2 restrict*).tw,kw. [mp=title, abstract, original title, name of substance word, subject heading word, floating sub-heading word, keyword heading word, protocol supplementary concept word, rare disease supplementary concept word, unique identifier, synonyms]

11 or/6-10

12 1 or 2 or 3 or 4

13 5 and 11 and 12

**Cochrane**

ID Search

#1 MeSH descriptor: [Diet] this term only

#2 MeSH descriptor: [Diet, Vegan] explode all trees

#3 MeSH descriptor: [Diet, Carbohydrate Loading] explode all trees

#4 MeSH descriptor: [Healthy Diet] explode all trees

#5 MeSH descriptor: [Diet, High-Protein] explode all trees

#6 MeSH descriptor: [Diet, High-Protein Low-Carbohydrate] explode all trees

#7 MeSH descriptor: [Diet, Diabetic] explode all trees

#8 MeSH descriptor: [Diet, Atherogenic] explode all trees

#9 MeSH descriptor: [Diet, Reducing] explode all trees

#10 MeSH descriptor: [Diet, Vegetarian] explode all trees

#11 MeSH descriptor: [Diet, High-Fat] explode all trees

#12 MeSH descriptor: [Diet, Fat-Restricted] explode all trees

#13 MeSH descriptor: [Diet, Protein-Restricted] explode all trees

#14 MeSH descriptor: [Diet, Mediterranean] explode all trees

#15 MeSH descriptor: [Diet, Carbohydrate-Restricted] explode all trees

#16 MeSH descriptor: [Diet, High-Fat] explode all trees

#17 MeSH descriptor: [Portion Size] explode all trees

#18 MeSH descriptor: [Serving Size] explode all trees

#19 MeSH descriptor: [Diet, Paleolithic] explode all trees

#20 MeSH descriptor: [Diet, Western] explode all trees

#21 (Diet or diets) near/2 (carbohydrate or diabetic or atherogenic or fat):ti,ab,kw (Word variations have been searched)

#22 (Diet or Diets) near/2 (Mediterranean or protein or reducing or vegetarian or vegan):ti,ab,kw (Word variations have been searched)

#23 (Diet or Diets) near/2 (macrobiotic or western or healthy or ketogenic or paleolithic):ti,ab,kw (Word variations have been searched)

#24 energ* near/2 intake:ti,ab,kw (Word variations have been searched)

#25 (Portion or serving) near/2 size:ti,ab,kw (Word variations have been searched)

#26 calori* near/2 restrict*:ti,ab,kw (Word variations have been searched)

#27 #1 or #2 or #3 or #4 or #5 or #6 or #8 or #9 or #10 or #11 or #12 or #13 or #14 or #15 or #16 or #17 or #18 or #19 or #20 or #21 or #22 or #23 or #24 or #25 or #26

#28 MeSH descriptor: [Obesity, Metabolically Benign] explode all trees

#29 MeSH descriptor: [Obesity, Morbid] explode all trees

#30 MeSH descriptor: [Obesity, Abdominal] explode all trees

#31 MeSH descriptor: [Overweight] explode all trees

#32 Obes*:ti,ab,kw (Word variations have been searched)

#33 Overweight*:ti,ab,kw (Word variations have been searched)

#34 #28 or #29 or #30 or #31 or #32 or #33

#35 #27 and #34

**Embase**

#39. #36 AND #37 AND #38

#38. #28 OR #29 OR #30 OR #31 OR #32 OR #33 OR #34 OR #35

#37. #1 OR #2 OR #3 OR #4 OR #5 OR #6 OR #7 OR #8 OR #9 OR #10 OR #11 OR #12 OR #13 OR #14 OR #15 OR #16 OR #17 OR #18 OR #19 OR #20 OR #21 OR #22 OR #23 OR #24 OR #25 OR #26 OR #27

#36. 'clinical trial'/de OR 'randomized controlled trial'/de OR 'randomization'/de OR 'single blind procedure'/de OR 'double blind procedure'/de OR 'crossover procedure'/de OR 'placebo'/de OR 'prospective study'/de OR ('randomi?ed controlled' NEXT/1 trial*) OR rct OR 'randomly allocated' OR 'allocated randomly' OR 'random allocation' OR (allocated NEAR/2 random) OR (single NEXT/1 blind*) OR (double NEXT/1 blind*) OR ((treble OR triple) NEAR/1 blind*) OR placebo*

#35. overweight*:ti,ab

#34. obes*:ti,ab

#33. 'sarcopenic obesity'/exp

#32. 'metabolic syndrome x'/exp

#31. 'metabolically benign obesity'/exp

#30. 'morbid obesity'/exp

#29. 'diabetic obesity'/exp

#28. 'abdominal obesity'/exp

#27. ((diet OR diets) NEAR/2 (diabetic OR atherogenic OR fat OR mediterranean OR protein OR reducing OR vegetarian OR vegan OR macrobiotic OR western OR healthy OR ketogenic OR paleolithic OR unhealthy OR calory OR healthy OR dash)):ti,ab

#26. ((serving OR portion) NEAR/2 size):ti,ab

#25. (calori* NEAR/2 restrict*):ti,ab #

24. (energ* NEAR/2 intake):ti,ab

#23. 'caloric restriction'/exp

#22. 'portion size'/exp

#21. 'caloric intake'/exp

#20. 'western diet'/exp

#19. 'vegetarian diet'/exp

#18. 'unhealthy diet'/exp

#17. 'raw food diet'/exp

#16. 'protein diet'/exp

#15. 'paleolithic diet'/exp

#14. 'mediterranean diet'/exp

#13. 'macrobiotic diet'/exp

#12. 'low carbohydrate diet'/exp

#11. 'low calory diet'/exp

#10. 'high fat/high sucrose diet'/exp

#9. 'cholesterol diet'/exp

#8. 'atherogenic diet'/exp

#7. 'high fiber diet'/exp

#6. 'healthy diet'/exp

#5. 'dash diet'/exp

#4. 'cereal-based diet'/exp

#3. 'carbohydrate loading diet'/exp

#2. 'carbohydrate diet'/exp

#1. 'high fat/high sucrose diet'/exp OR 'high fat/high sucrose diet'

1. **Supplementary Tables and Figures**

**Table S1. Summary of the obesity guidelines identified on the National Guideline Clearinghouse (**[**www.guideline.gov/**](http://www.guideline.gov/)**)**

| **Guideline**  **Institution/Society**  **Year Reference** | **Recommendation for dietary changes** |
| --- | --- |
| **Screening for and management of obesity in adults: U.S. Preventive Services Task Force Recommendation Statement**  **2012 (Moyer, 2012)** | No specific recommendations |
| **Diagnosis and management of obesity**  **American Academy of Family Physicians 2013 (Physicians, 2013)** | Several guidelines adopted. Healthy eating following MyPlate, or caloric restriction as per the National Heart, Lung and Blood Institute |
| **Obesity prevention and management**  **University of Michigan Health System**  **2013** | *Appropriate portion sizes of whole grains, fruits and vegetables, and lean meats/dairy* [Grade I D]  *Decrease intake of high calorie foods and drinks, including alcohol* [Grade I A]  *Stay within your daily calorie needs or use the plate method to control caloric intake* |
| **Clinical practice guidelines for the management of overweight and obesity in adults, adolescents and children in Australia.**  **National Health and Medical Research Council**  **2013 (Council, 2013)** | *Current Australian Dietary Guidelines should be used as the basis of advice on nutrition for adults.*  *For adults who are overweight or obese, design dietary interventions for weight loss to produce a 2,500 kilojoule per day energy deficit and tailor programs to the dietary preferences of the individual* (Grade A) |
| **2013 American Heart Association/ American College of Cardiology/The Obesity Society guideline for the management of overweight and obesity in adults: a report of the American College of Cardiology/American Heart Association Task Force on Practice Guidelines and The Obesity Society.**  **2013 (Jensen et al., 2014)** | *-Prescribe a diet to achieve reduced calorie intake for obese or overweight individuals who would benefit from weight loss, as part of a comprehensive lifestyle intervention. Any one of the following methods can be used to reduce food and calorie intake:* NHLBI Grade: A (Strong); ACC/AHA COR: I; ACC/AHA LOE: A  -*Prescribe 1,200–1,500 kcal/d for women and 1,500–1,800 kcal/d for men (kilocalorie levels are usually adjusted for the individual's body weight)*  *-Prescribe a 500-kcal/d or 750-kcal/d energy deficit*  *-Prescribe one of the evidence-based diets that restricts certain food types (such as high-carbohydrate foods, low-fiber foods, or high-fat foods) in order to create an energy deficit by reduced food intake.*  *-Prescribe a calorie-restricted diet for obese and overweight individuals who would benefit from weight loss, based on the patient's preferences and health status, and preferably refer to a nutrition professional for counseling. A variety of dietary approaches can produce weight loss in overweight and obese adults, as presented in critical question (CQ) 3, evidence statement (ES) 2.* NHLBI Grade: A (Strong) |
| **VA/DoD clinical practice guideline for screening and management of overweight and obesity.**  **Department of Veterans Affairs, Department of Defense**  **2014 (Affairs, 2014)** | *Offer any of several diets that produce a caloric deficit and have evidence for weight loss efficacy and safety (e.g., low-carbohydrate, Dietary Approaches to Stop Hypertension [DASH], or low-fat)* [Grade A] |
| **Obesity: identification, assessment and management of overweight and obesity in children, young people and adults**  **National Institute for Health and Care Excellence (NICE)**  **2014 (Excellence, 2014)** | Diets that have a 600 kcal/day deficit (that is, they contain 600 kcal less than the person needs to stay with the same weight) or that reduce calories by lowering the fat content (low-fat diets), in combination with expert support and intensive follow-up, are recommended for sustainable weight loss. |
| **Pharmacological management of obesity: An Endocrine Society Clinical Practice Guideline**  **2015 (Apovian et al., 2015)** | Management is recommended through caloric restriction, diet and behavior modification |
| **American Association of Clinical Endocrinologists/American College of Endocrinology comprehensive clinical practice guidelines for medical care of patients with obesity**  **2016 (Garvey et al., 2016)** | *Reducing total energy (caloric) intake should be the main component of any weight-loss intervention* (Grade A; BEL 1)  *Even though the macronutrient composition of meals has less impact on weight loss than adherence rates in most patients, in certain patient populations, modifying macronutrient composition may be considered to optimize adherence, eating patterns, weight loss, metabolic profiles, risk factor reduction, and/or clinical outcomes* (Grade A; BEL 1) |
| **POWER — Practice Guide on Obesity and Weight Management, Education, and Resources American Gastroenterology Association**  **2017 (Acosta et al., 2017)** | Reduced diet (without further details) |
| **European practical and patient centered guidelines for adult obesity management in primary care**  **2019 (Schutz et al., 2019)** | *Decrease energy density of food;*  *increase vegetables and eat two portions of fruit per day;*  *decrease fatty foods, especially saturated fat;*  *decrease refined carbohydrates, sugar and sweetened beverages;*  *decrease portion size, use a smaller plate and eat only one portion per meal.*  *Avoid snacking and skipping meals (breakfast, for example: if you are not hungry early in the morning, you can eat your breakfast later, when you will feel hungrier)* |
| **Canadian Medical Association**  **obesity in adults: a clinical practice guideline**  **2020 (Wharton et al., 2020)** | A healthy, well-balanced eating pattern.  Weight loss and weight-loss maintenance require a long-term reduction in caloric intake.  *We suggest that nutrition recommendations for adults of all body sizes be personalized to meet individual values, preferences and treatment goals to support a dietary approach that is safe, effective, nutritionally adequate, culturally acceptable and affordable for long-term adherence*. Grade D; level 4 |

Abbreviations: BEL: Best evidence level

Sentences in italic are taken verbatim from the guidelines

**Table S2: Summary of the included randomized trials included**

| **First Author, Country, Protocol Number (if available)** | **Diet brands (categories)** | **Funding body** | **Physical Activity (Yes/No)** | **Behavioral Support (Yes/No)** | **Intervention Duration (months)** | **n randomized/**  **n completed** | **Age Mean (SD) (years)** | **Men (%)** | **Menopausal Status and/or Co-morbidities (%)** | **Baseline BMI Mean (SD) (kg/m^2^)** |
| --- | --- | --- | --- | --- | --- | --- | --- | --- | --- | --- |
| Marniemi, Finland (Marniemi et al., 1990) | Lactovegetarian (MM) | N/A | No | No | 12 | N/A/31 | 38 (N/A) | 26 | None | 34.4 |
|  | Mixed (MM) |  |  |  | 12 | N/A /37 | 38 (N/A) | 27 | None | 33.6 |
| Williams, USA (Williams et al., 1994) | Diet (MM) | National Heart, Lung, and Blood Institute of the National Institutes of Health, National Dairy Promotion and Research Board | No | No | 12 | 45/39 | N/A | 100 | N/A | 30.4 (3.4) |
|  | Control (UD) |  |  |  | 12 | 44/40 | N/A | 100 | N/A | 30.7 (2.2) |
| Knopp, USA  (Knopp et al., 1997) | Diet 1, 30% fat (MM) | National Institutes  of Health, the Clinical Nutrition Research Unit and the Diabetes  and Endocrinology Research Center grant at the University of Washington, and a gift from the Robert B. McMillen family trust | No | Yes | 12 | 78/N/A | 46 (10) | 100 | HL: 100% | 26.3 (3.2) |
|  | Diet 2, 26% fat (MM) |  |  |  | 12 | 62/N/A | 48 (8) | 100 | HL: 100% | 25.9 (2.5) |
|  | Diet 3, 22% fat (MM) |  |  |  | 12 | 71/N/A | 46 (9) | 100 | HL: 100% | 26 (2.9) |
|  | Diet 4, 18% fat (LFHC) |  |  |  | 12 | 59/N/A | 49 (10) | 100 | HL: 100% | 26 (3.2) |
| Pritchard, Australia (Pritchard et al., 1997) | Diet (MM) | Victorian Health Promotion Foundation, the William Buckland Foundation and the Department of Medicine, University of Melbourne | No | Yes | 12 | 22/18 | 44 (6) | 100 | N/A | 29 (2.8) |
|  | Control (UD) |  |  |  | 12 | 20/19 | 42 (4.5) | 100 | N/A | 28.6 (2.8) |
| Wing, USA  (Wing et al., 1998) | Exercise (Usual diet) | National Institute of Health, the Obesity /Nutrition Research Center and the General Clinical Research Center | Yes | Yes | 12 | 37/28 | 46 (4.5) | 19 | N/A | 36.1 (4.1) |
|  | Low fat with exercise (LFHC) |  |  |  | 12 | 40/31 | 46 (3.8) | 23 | N/A | 35.7 (4.1) |
| Due, Denmark  (Due et al., 2004) | Medium Protein (MM) | The Danish Research and Development Program for Food Technology, the Federation of Danish Pig Producers and Slaughterhouse, Danish Dairy Research Foundation and The Danish Livestock and Meat Board. Some food companies contributed to the food selection | No | Yes | 12 | 25/18 | 39 (35–44) | 24 | N/A | 30.8 (29.9–31.6) |
|  | High Protein (MM) |  |  |  | 12 | 25/23 | 40 (36–448) | 24 | N/A | 30.0 (29.1–30.9) |
| Pereira, USA  (Pereira et al., 2004) | Low-Fat (LFHC) | National Institute of Diabetes and Digestive and Kidney Diseases National Institutes of Health, and grants from General Mills Corporation and Charles H. Hood Foundation | No | Yes | 24 | 23/17 | 33 (4.3) | 24 | N/A | 33 (4.6) |
|  | Low–Glycemic Load (HFLC) |  |  |  | 24 | 23/22 | 29 (6.3) | 23 | N/A |  |
| Stern, USA  (Stern et al., 2004) | Low Carbohydrate (HFLC) | The Veterans Affairs Healthcare Network Competitive Pilot Project Grant | No | No | 12 | 64/44 | 53 (9) | 82 | CHD: 16%  Depression: 33%  DM: 42%  HTN: 72%  DL: 51%  Smokers: 20% | 43.6 (6.9) |
|  | Conventional Diet (MM) |  |  |  | 12 | 68/43 | 54 (9) | 81 | CHD: 16%  Depression: 34%  DM: 40%  HTN: 57%  DL:50%  Smokers: 22% | 42.3 (5.9) |
| Dansinger, USA (Dansinger et al., 2005) | Atkins (HFLC) | General Clinical Research Center via the National Center for Research Resources of the National Institutes of Health, the US Department of Agriculture, the Human Metabolic and Genetics Core Laboratory of the Boston Obesity Nutrition Research Center program, the Agency for Healthcare Research and Quality | Yes | No | 12 | 40/N/A | 47 (12) | 47 | Fating glucose ≥110 mg/dl: 40% | 35 (3.5) |
|  | Zone (HFLC) |  |  |  | 12 | 40/N/A | 51 (9) | 50 | Fating glucose ≥110 mg/dl: 20% | 34 (4.5) |
|  | Weight Watchers (MM) |  |  |  | 12 | 40/N/A | 49 (10) | 42 | Fating glucose ≥110 mg/dl: 20% | 35 (3.8) |
|  | Ornish (LFHC) |  |  |  | 12 | 40/N/A | 49 (12) | 57 | Fating glucose ≥110 mg/dl: 30% | 35 (3.9) |
| Ebbeling, USA (Ebbeling et al., 2005) | Low Glycemic Load (MM) | National Institute of Diabetes and Digestive Kidney Diseases, the Charles H Hood Foundation, National Institutes of Health to support the General Clinical Research Center at Children’s Hospital Boston | Yes | Yes | 12 | 17/11 | 29.8 (7) | 12 | Healthy | N/A |
|  | Low-fat (MM) |  |  |  | 12 | 17/12 | 27.2 (5.4) |  | Healthy | N/A |
| Azadbakht, Iran (Azadbakht et al., 2007) | Moderate fat (MM) | N/A | No | No | 14 | 50/45 | 45(5) | 32 | Healthy | 29 (10) |
|  | Low-fat (LFHC) |  |  |  | 14 | 50/44 | 46(6) | 25 | Healthy | 29.2 (11) |
| Gardner, USA  NCT00079573(Gardner et al., 2007) | Atkins (HFLC) | National Institutes of Health, Community Foundation of Southeastern Michigan, and by Human Health Service, General Clinical Research Centers, National Center for Research Resources, National Institutes of Health | No | Yes | 12 | 77/68 | 42 (5) | 0 | MetS 29% | 32 (4) |
|  | ZONE (HFLC) |  |  |  | 12 | 79/61 | 40 (6) | 0 | MetS 25% | 31 (3) |
|  | Learn (MM) |  |  |  | 12 | 79/61 | 40 (7) | 0 | MetS 37% | 31 (4) |
|  | Ornish (LFHC) |  |  |  | 12 | 76/59 | 42 (6) | 0 | MetS 36% | 32 (3) |
| Keogh, Australia  N012605000614695 (Keogh et al., 2007) | High monounsaturated fat (HFLC) | National Health and Medical Research Council of Australia | No | No | 12 | Total n randomized=73/n completed= 19/group | 52 (8) | N/A | Hyperinsulinemic: 100% | 34 (4) |
|  | High-Protein (HFLC) |  |  |  | 12 |  | 48 (13) | N/A | Hyperinsulinemic: 100% | 34 (4) |
| Salas-Salvado, Spain  ISRCTN35739639  (Salas-Salvadó et al., 2008) | Mediterranean diet-extra virgin olive oil (HFLC) | The Spanish Ministry of Health and grants and from the Generalitat Valenciana. | No | No | 12 | 419/409 | 67 (5.9) | 45 | HTN: 81% DM2: 47% HL: 64% MetS: 62% | 29.2 (3.1) |
|  | Mediterranean diet-nuts (HFLC) |  |  |  | 12 | 423/411 | 67 (5.7) | 51 | HTN: 81% DM: 46% HL: 62.3% MetS: 61% | 29.3 (3.2) |
|  | Control diet (MM) |  |  |  | 12 | 422/404 | 68 (6.2) | 43 | DM: 46%  HTN: 81% HL:67% MS: 62% | 29.5 (3.5) |
| Shai, Israel  NCT00160108 (Shai et al., 2008) | Low-fat (MM) | Nuclear Research Center Negev, the Dr. Robert C. and veronica Atkins Research Foundation , and the S. Daniel Abraham International Center for Health and Nutrition, Ben-Gurion University, Israel. | No | No | 24 | 104/94 | 51 (7) | 86 | CHD: 37%  DM: 12% HL: 27% HTN: 22% | 30.6 (3.2) |
|  | Mediterranean (MM) |  |  |  | 24 | 109/93 | 53 (6 ) | 82 | CHD: 42%  DM: 14% HL: 27% HTN: 34% | 31.2 (4.1) |
|  | Low-carb (HFLC) |  |  |  | 24 | 109/85 | 52 (7) | 91 | CHD: 31%  DM2: 17% HL: 25% HTN: 33% | 30.8 (3.5) |
| Dale, New Zealand  NCT00128336  (Dale et al., 2009) | High-monounsaturated-fat (HFLC) | Health Research Council of New Zealand | Yes | Yes | 24 | 100/85 | 45 (10) | 0 | N/A | 31.9 (4.9) |
|  | High-carbohydrate (MM) |  |  |  | 24 | 100/89 | 45 (11) | 0 | N/A | 31.8 (5.2) |
| Frisch, Germany  NCT00868387  (Frisch et al., 2009) | Low fat (MM) | Different German health insurance companies and the 'Institute for Applied Telemedicine', Heart Center NRW, Ruhr University Bochum, Bad Oeynhausen, Germany | No | No | 12 | 100/80 | 47 (11) | 24 | DM: 1%  HTN: 31%  HL: 4% | 33.8 (4.8) |
|  | Low Carbohydrate (HFLC) |  |  |  | 12 | 100/85 | 47 (10.3) | 38 | DM: 4%  HTN: 24% HL: 6% | 33.5 (3.9) |
| Tanumihardjo, USA (Tanumihardjo et al., 2009) | High vegetable (MM) | National Research Initiative of the USDA Cooperative State Research, Education, and Extension Service and the Wisconsin Cranberry Board | Yes | No | 12 | 30/14 | 31 (6.6) | 27 | N/A | 33.7 (3.8) |
|  | Energy and fat-reduction diet (MM) |  |  |  | 12 | 30/18 | 36 (9.4) | 27 | N/A | 33.3 (3.5) |
| Klemsdal, Norway (Klemsdal et al., 2010) | Low glycemic load diet (HFLC) | Norwegian National Research Council | No | No | 12 | 100/78 | 50 (9.3) | 46 | HTN: 35%  MetS: 65% | N/A |
|  | Low-fat diet (MM) |  |  |  | 12 | 102/86 | 50 (8.4) | 38 | HTN: 28%  MetS: 60% | N/A |
| Sukumar, USA  NCT00473031 (Sukumar et al., 2011) | High Protein (MM) | National Institutes of Health and a Busch Biomedical Award | No | Yes | 12 | 29/26 | 59 (4.1) | 0 | Postmenopausal: 100% | 32.1 (4.6) |
|  | Normal Protein (MM) |  |  |  | 12 | 31/21 | 57 (4.7) | 0 | Postmenopausal: 100% |  |
| Venn, New Zealand (Venn et al., 2010) | Pulse and wholegrain diet (MM) | New Zealand Foundation for Research, Science, and Technology, and the Lifestyle Foods program industry partners. | No | No | 18 | 53/43 | 42 (11) | 16 | HTN: 10% | 36.1 (6.5) |
|  | Control Group (MM) |  |  |  | 18 | 55/30 | 42 (10) | 12 | HTN: 12% | 34.7 (4.6) |
| Foster-Schubert, USA (Foster‐Schubert et al., 2012) | Diet with exercise (MM) | National Cancer Institute and National Institute of Health grants | Yes | Yes | 12 | 117/108 | 58 (4.5) | 0 | Postmenopausal: 100% | 31.0 (4.3) |
|  | Control diet and exercise (UD) |  |  |  | 12 | 117/106 | 58 (5.0) | 0 | Postmenopausal: 100% | 30.7 (3.7) |
| De Jonge, USA  NCT00072995  (De Jonge et al., 2012) | Low-Fat, Average Protein (LFHC) | National Heart, Lung, and Blood Institute and the General Clinical Research Center, National Institutes of Health. | Yes | Yes | 24 | 204/123 | 51 (8.9) | 38 | N/A | 32.6 (3.7) |
|  | Low-Fat, High Protein (LFHC) |  |  |  | 24 | 202/128 | 51 (9.5) | 33 | N/A | 32.6 (13.7) |
|  | High-Fat, Average Protein (HFLC) |  |  |  | 24 | 204/114 | 58 (9.3) | 4 | N/A | 32.3 (83.9) |
|  | High-Fat, High Protein (HFLC) |  |  |  | 24 | 201/132 | 51 (9.1) | 36 | N/A | 33.2 (4.2) |
| Fernandez,  Spain  (Fernández et al., 2012) | Insulin Resistant, “Diet A” (HFLC) |  | No | No | 12 | 13/4 | 36 (12) | 46 | N/A | 31.2 (2.7) |
|  | Insulin Resistant, “Diet B” (MM) |  |  |  | 12 | 12/4 | 42 (15) | 25 | N/A | 32 (2.0) |
|  | Insulin Sensitive, “Diet A” (HFLC) |  |  |  | 12 | 8/4 | 47 (12) | 12.5 | N/A | 33 (2.5) |
|  | Insulin Sensitive, “Diet B” (MM) |  |  |  | 12 | 7/4 | 41 (15) | 42.8 | N/A | 31.4 (2.2) |
| Wycherley, Australia  ACTRN12606000002583  (Wycherley et al., 2012) | High Protein (HFLC) | Meat and Livestock Australia | No | No | 12 | 58/33 | 51 (9.4) | 100 | N/A | 33 (3.9) |
|  | High Carbohydrate (MM) |  |  |  | 12 | 62/35 | 50 (9.3) | 100 | N/A |  |
| Griffin, Australia  ACTRN12609000307202 (Griffin et al., 2013) | Higher Protein (HFLC) | Meat and Livestock Australia | Yes | Yes | 12 | 36/21 | 22 (22 - 23) | 0 | N/A | 34.1 (32.7 - 35.5) |
|  | Higher Carbohydrate (MM) |  |  |  | 12 | 35/15 | 23 (22 - 23) | 0 | N/A | 34.3 (32.8 - 35.5) |
| Jesudason, USA  ACTRN12608000229370(Jesudason et al., 2013) | High Protein (MM) | N/A | No | No | 24 | 164/69 | 59 (0.6) | 0 | Postmenopausal: 100% | 34.6 (0.5) |
|  | High Normal protein (MM) |  |  |  | 24 | 159/67 | 59 (0.6) | 0 | Postmenopausal: 100% | 33.7 (0.5) |
| Rusu, Romania  (Rusu et al., 2013) | Normoglucidic low-calorie (MM) | Romanian National Authority for Scientific Research | Yes | No | 12 | 60/58 | 54 (8.6) | 45 | DM: 31%,  HTN: 48% Hypertiglyceridemia: 57 %  MetS: 62% | 29.4 (3.5) |
|  | Low Fat (LFHC) |  |  |  | 12 | 60/52 | 54 (9.3) | 40 | DM: 29%  HTN: 58% Hypertriglyceridemia: 65%  MetS: 62% | 29.4 (3.4) |
| Bazzano, USA  NCT00609271  (Bazzano et al., 2014) | Low-fat (MM) | National Center for Research Resources of the National Institutes of Health | No | Yes | 12 | 73/60 | 48 (10) | 11 | HL: 12%  HTN: 33% | 35.6 (4.5) |
|  | Low-carbohydrate (HFLC) |  |  |  | 12 | 75/59 | 46 (9.9) | 12 | HL: 16%  HTN: 28% | 35.2 (3.8) |
| Mellberg, Sweden  NCT00692536  (Mellberg et al., 2014) | Low-carbohydrate (HFLC) | The Swedish Council for Working Life and Social Research, the Swedish Research Council, the Swedish Heart and Lung Foundation, the County Council of Va ̈sterbotten and Umeå University, Sweden. | No | Yes | 12 | 75/59 | 46 (9.9) | 0 | HTN: 28.0% HL: 16% | 35.2 (3.8) |
|  | Paleolithic Diet (MM) |  |  |  | 24 | 35/27 | 60 (5.5) | 0 | Postmenopausal: 100% | 32.7 (3.6) |
| Ma, USA  [NCT00911885](https://clinicaltrials.gov/ct2/show/NCT00911885)  (Ma et al., 2015) | High Fiber Diet (MM) | National Heart, Lung and Blood Institute | No | No | 12 | 121/100 | 52 (10) | 33 | MetS: 100% | 35 (2.8) |
|  | AHA Diet (MM) |  |  |  | 12 | 119/104 | 53 (9.9) | 34 | MetS: 100% | 34.9 (3.1) |
| Santanasto, USA  NCT00714506 (Santanasto et al., 2015) | Diabetes Prevention Program (MM) | N/A | Yes | No | 12 | 21/15 | 71 (5.9) | 19.1 | n/a | 33.6 (3.3) |
|  | Control (Usual diet) |  |  |  | 12 | 18/14 | 70 (6.2) | 14.3 | n/a | 32 (3.1) |
| Brinkworth, Australia (Brinkworth et al., 2016) | Very low carbohydrate, high fat (HFLC) | National Heart Foundation of Australia and the National Health and Medical Research Council of Australia | No | No | 12 | N/A/32 | 52 (7.8) | 31.2 | N/A | 33.7 (4) |
|  | Higher carbohydrate, low fat (MM) |  |  |  | 12 | N/A/33 | 51 (6.5) | 39 | N/A | 33.2 (4) |
| Gepner, Israel  NCT01530724 (Gepner et al., 2017) | Low-fat (MM) | The Deutsche Forschungsgemeinschaft, the Israel Science Foundation, the Israel Ministry of Science and Technology, and the Dr Robert C. and Veronica Atkins Research Foundation | Yes | No | 18 | 139/118 | 48 (9.2) | 87.7 | HL: 12% HTN: 8%  Hyperglycemia: 4% | 30.8 (3.7) |
|  | Mediterranean (HFLC) |  |  |  | 18 | 139/122 | 47 (9.3) | 89.9 | HL: 12% HTN: 8% Hyperglycemia: 4% | 30.9 (4.0) |
| Gardner, USA  NCT01826591(Gardner et al., 2018) | Low-Fat (MM) | National Institute of Diabetes and Digestive and Kidney Diseases, Nutrition Science Initiative, National Heart, Lung, and Blood Institute, and the Stanford Clinical and Translational Science Award | Yes | Yes | 12 | 314/241 | 39 (6.8) | 45.2 | MetS: 35% | 33.4 (3.4) |
|  | Low-Carbohydrate (HFLC) |  |  |  | 12 | 318/238 | 40 (6.7) | 41.1 | MetS: 33% | 33.3 (3.4) |
| Pavic, Croatia  NCT02259244  (Pavić et al., 2019) | Mediterranean (HFLC) | N/A | Yes | Yes | 12 | 63/40 | 46 (13) | 30.2 | Depression:9.5%  MetS: 68%  Smokers: 16% | 40.6 (6.7) |
|  | Standard hypolipemic (MM) |  |  |  | 12 | 61/44 | 49 (12) | 21.2 | Depression:9.8%  MetS: 67%  Smokers: 19% | 40.4 (6.4) |

CHD: coronary heart disease, DM: Diabetes Mellitus, HL: Hyperlipidemia, HFLC: High Fat Low Carbohydrate, HTN: Hypertension, LFHC: Low Fat High Carbohydrate, MetS: Metabolic Syndrome; MM: Moderate Macronutrients, N/A: information not available

**Table S3: Risk of bias of the randomized controlled trials included in the systematic review, using the Cochrane risk of bias assessment tool**

| **First Author, Country** | **Random Sequence Generation** | **Allocation Concealment** | **Blinding of participants & personnel** | **Blinding of outcome assessment** | **Incomplete outcome data** | **Selective reporting** | **Other Bias** |
| --- | --- | --- | --- | --- | --- | --- | --- |
| Marniemi, Finland (Marniemi et al., 1990) | *“136 persons were randomly assigned according to sex, age and overweight to three groups…”*  Method of randomization is unclear | No description | No description of blinding of participants and dieticians | No description | No dropout | No published protocol | No description of baseline characteristics, COI and funding bodies. |
|  | Unclear | Unclear | Unclear | Unclear | Low | Unclear | Unclear |
| Williams, USA (Williams et al., 1994) | Assigned at random, no additional details | No description | No description of blinding of participants and dieticians | Outcome assessor not described | No description | No published protocol | No imbalance in baseline characteristics.  COI and funding: No description |
|  | Unclear | Unclear | Unclear | Unclear | Unclear | Unclear | Unclear |
| Knopp, USA (Knopp et al., 1997) | No description | No description | No description of blinding of participants and dieticians | No description | 47 dropped out (overall rate is 10.6%); Reasons for dropout are available, but not evenly distributed across arms | No published protocol | Imbalance in some baseline characteristics (smokers, alcohol consumers and education level)  COI: no description  No industrial sponsorship. |
|  | Unclear | Unclear | Unclear | Unclear | High | Unclear | High |
| Pritchard, Australia (Pritchard et al., 1997) | No description | No description | No description of blinding of participants and dieticians | No description | Drop-out rate ranged was in the range of 5-19%, with significant differences across groups | No published protocol | No imbalance in baseline characteristics  No COI  No industrial sponsorship |
|  | Unclear | Unclear | Unclear | Unclear | High | Unclear | Low |
| Wing, USA (Wing et al., 1998) | No description | No description | No description of blinding of participants or dieticians or other personnel (behavior therapist, exercise physiologist) | No description | Drop out variable across arms, ranging from 11 to 25%.  Pooled calculation showed a dropout rate 22% | No published protocol | No imbalance in baseline characteristics  No COI  No industrial sponsorship |
|  | Unclear | Unclear | Unclear | Unclear | High | Unclear | Low |
| Due, Denmark (Due et al., 2004) | No description | No description | The energy content was not revealed to the participants.  Blinding of dieticians not described.  No information on other personnel | No description | Drop-out rate ranged was in the range of 8-28%, with significant differences across groups | No published protocol | No imbalance in baseline characteristics, no COI. Sponsorship by food manufacturers |
|  | Unclear | Unclear | Low | Unclear | High | Unclear | High |
| Pereira,USA (Pereira et al., 2004) | “*Sequence was randomly generated by computer*” | ”*Envelopes were prepared separately for male and female participants, numbered sequentially, beginning with one, enclosed with dietary group assignment, and sealed until randomization*” | Participants, dietitians, and personnel not blinded | *“Study personnel who measured the main process measure (body weight) and the main end point (REE) were masked”* | Dropout ranged between 4 and 26% per arm. | No published protocol | Imbalance in some baseline characteristics (age and ethnicity).  No COI  No industrial sponsorship |
|  | Low | Low | High | Low | High | Unclear | High |
| Stern, USA (Stern et al., 2004) | “*Randomization was performed by using a pre-established algorithm generated from a random set of numbers that was constructed and held in a separate center and concealed from those enrolling persons during randomization*” | *“Randomization was performed by using a pre-established algorithm generated from a random set of numbers that was constructed and held in a separate center and concealed from those enrolling persons during randomization”* | No description of blinding of participants and dieticians | No description | 34% dropout from both study arms | No published protocol | No imbalance in baseline characteristics  No COI  No industrial sponsorship  , |
|  | Low | Low | Unclear | Unclear | High | Unclear | Low |
| Dansinger, USA (Dansinger et al., 2005) | *“Once each of the 4 class rosters contained approximately 10 participants, 1 of the 4 diets was assigned to each group according to a computer-generated randomized Latin-square sequence*” | *“Study personnel were blinded to dietary assignments (revealed by the study statistician) until after each class roster was finalized, to avoid the potential for biased recruiting according to diet type*» | Participants not blinded  *“At the first meeting, the team revealed the diet assignment and provided the corresponding rationale”*  Dieticians not blinded  *“A single team composed of a dietitian and physician (M.L.D., J.A.G.) administered diet-specific advice to each group, meeting for 1 hour on 4 occasions during the first 2 months of the study»*  No mention of physician’s blinding. | “*Study nurses and laboratory personnel who assessed outcomes were blinded to participants’ dietary assignment*”. It is not clear whether they were involved in the measurements of anthropometrics | 35-50% dropout | No published protocol | No imbalance in baseline characteristics  No COI  No industrial sponsorship |
|  | Low | Low | Unclear | Unclear | High Risk | Unclear | Low |
| Ebbeling, USA (Ebbeling et al., 2005) | *“Subjects were randomly assigned to the experimental (low-GL diet) or conventional (low-fat diet) treatment group between August 2001 and July 2002”,* but no further details. | No description | No description of blinding of participants and dieticians | No description | 29-35% dropout; imputation methods not described | No published protocol | No imbalance in baseline characteristics  No COI.  Funding: No description |
|  | Unclear | Unclear | Unclear | Unclear | High | Unclear | Unclear |
| Azadbakht, Iran (Azadbakht et al., 2007) | “*Forty-five subjects were randomized using a program generated by a random number table to an energy-controlled diet containing 30 % energy as fat, and forty-four subjects to an energy-controlled diet containing 20 % energy as fat”* | No description | Dieticians and participants not blinded  *“All subjects were given general oral and written information about healthy food choices and a diet 2090 kJ (500 kcal) below their energy needs according to their weight, which was offered according to specific individualized programs at baseline and at subsequent visits”*  *“The nutritionist who prescribed the diets had to be aware of the group assignment”* | *“Laboratory staff were not aware to which group the patients had been assigned”*  Blinding of assessors of anthropometrics not described | 10-12% dropout rate | No published protocol | No imbalance in baseline characteristics.  COI and funding: No description |
|  | Low | Unclear | Unclear | Unclear | Low | Unclear | Unclear |
| Gardner, USA  (Gardner et al., 2007) | Randomization in blocks of 24 (6 per treatment group) but no description on the actual method. | *“A blinded research technician selected folded pieces of paper with group assignments from an opaque envelope”* | Participants and dieticians not blinded. | Clinic and laboratory staff were blinded | 22% dropout per arms. Used ITT but no description of imputation methods | Registered protocol: NCT00079573.  Outcomes pre-specified | No imbalance in baseline characteristics  No COI  No industrial sponsorship |
|  | Unclear | Low | Unclear | Low | High | Low | Low |
| Keogh, Australia  (Keogh et al., 2007) | No description of sequence generation method | No description | No description | No description | Dropout rate:48% | No published protocol | Imbalance in some baseline characteristics (age and insulin).  No COI  No industrial sponsorship. |
|  | Unclear | Unclear | Unclear | Unclear | High | Unclear | High |
| Salas-Salvado, Spain  (Salas-Salvadó et al., 2008) | A computer generated random number sequence was used for sequence generation. Yet, departures from the protocol were later revealed, including assigning 425 participants from the same household to the same diet.  Serious deviation from protocol. Study was retracted and then republished after excluding patients who have not been properly randomized | At the start of the study, randomization was concealed employing closed envelopes. Yet, sealed envelopes were not used all throughout. | No description of blinding of participants and dieticians | No description | Dropout 2-4% with no differences between groups. | Registered protocol: ISRCTN35739639.  Outcomes pre-specified. | No imbalance in baseline characteristics  No COI  No industrial sponsorship |
|  | High | High | Unclear | Unclear | Low | Low | Low |
| Shai, Israel  (Shai et al., 2008) | *“The participants were randomly assigned with- in strata of sex, age (below or above the median), BMI (below or above the median), history of coronary heart disease (yes or no), history of type 2 diabetes (yes or no), and current use of statins (none, <1 year, or ≥1 year) with the use of Monte Carlo simulations”* | No description | No description of blinding of participants and dieticians | No description | 10-23% dropout rate; dropout not evenly distributed across arms | Registered protocol: NCT00160108. Outcomes pre-specified | No imbalance in baseline characteristics  No COI  No industrial sponsorship |
|  | Unclear | Unclear | Unclear | Unclear | High | Low | Low |
| Dale, New Zealand  (Dale et al., 2009) | Randomly assigned using block randomization but no further details on sequence generation were provided | *“We randomly assigned the first 200 women who met the study criteria in blocks of 20 using numbered, opaque sealed envelopes, after stratification based on the extent of their weight loss (5%–7% or > 7% of initial body weight) using a 2 X2 factorial design. Each envelope contained a card that described the nature of the intervention”* | Participants were blinded.  “In accordance with the informed consent form, participants in each group were aware that they had been allocated to groups involving varying levels of support and different types of dietary advice, but they were not provided with details of the alternative program or diets »  Dietician and other personnel not blinded.  Other personnel not blinded.  “*The same senior registered nurse, experienced research dietician, nutritionist and exercise trainer provided the intervention advice throughout the study, made the clinical measurements (having had formal training in anthropometry) and took the blood samples. Although it was clearly not possible for the personnel to be masked as to group allocation”* | *“The same senior registered nurse, experienced research dietician, nutritionist and exercise trainer provided the intervention advice throughout the study, made the clinical measurements (having had formal training in anthropometry) and took the blood samples. Although it was clearly not possible for the personnel to be masked as to group allocation****,*** *the research assistants and laboratory technicians responsible for analysis of diet records and blood samples were unaware of the program and diet groups”*  Outcome assessors for anthropometrics were not blinded | 20% dropout per arm, reasons for dropout described and methods for imputation described | Registered protocol: NCT00128336. Outcomes pre-specified | No imbalance in baseline characteristics  No COI  No industrial sponsorship |
|  | Unclear | Low | High | High | Low | Low | Low |
| Frisch, Germany  (Frisch et al., 2009) | “*Participants (n = 200) were randomly assigned by computer-generated random number lists in two equal groups*” | No description | No description of participants and personnel. | No description | 15-20% dropout rate, reasons for dropout described and balanced across both groups; ITT was used and in case of missing data imputation was done using baseline characteristics | Registered protocol: NCT00868387. Outcomes pre-reported | One imbalance in baseline characteristics (gender).  No COI.  Funded by company for telehealth, not related to diets. |
|  | Low | Unclear | Unclear | Unclear | Low | Low = | High |
| Sacks, USA (Sacks et al., 2009) | Random assignment was generated by the data manager. Method used not described. | No description | Participants and dieticians not blinded  *“Staff and participants were taught that each diet adhered to principles of a healthful diet and that each had been recommended for long-term weight loss, thereby establishing equipoise.”* | Investigators and staff who assessed outcomes were blinded to the diet assignment. | 20% dropout, evenly distributed across arms, but described | Registered protocol NCT00072995, outcomes pre-specified | No imbalance in baseline characteristics  No COI  No industrial sponsorship |
|  | Unclear | Unclear | Low | Low | Low | Low | Low Risk |
| Tanumihardjo, USA (Tanumihardjo et al., 2009) | Participants were randomized, but no other details | No description | No description of participants blinding  Researchers assisting in dietary advice were not blinded  No additional personnel mentioned | Researchers measuring weight and body composition were not blinded. Those measuring blood samples were blinded | 20-30% dropout rate and missing values were disregarded | No published protocol | Imbalance in age at baseline  Funding and COI: No description |
|  | Unclear | Unclear | Unclear | High | High | Unclear | High |
| Klemsdal, Norway (Klemsdal et al., 2010) | Randomized participants but no further description included | No description | No description of blinding of participants and dieticians | No description | Dropout rate was 22% in the Low Glycemic Load (LGL) group and 16% in the Low Fat group  However, attendance rate for the total study population at 3, 6, 9 and 12 months was 94%, 86%, 77% and 82%, respectively, with no significant between group differences. | No published protocol | No imbalance in baseline characteristics.  No COI.  No mention of funding source |
|  | Unclear | Unclear | Unclear | Unclear | Low | Unclear | Unclear |
| Sukumar, USA  (Sukumar et al., 2011) | Randomization was done using SAS program: | No description | No description of blinding of participants and dieticians | No description | Drop-out rate ranged was in the range of 10-32%, with significant differences across groups | Registered protocol: NCT00473031. Weight, BMI and waist circumference not mentioned in pre-specified outcomes. | Imbalance in some baseline characteristics (age and weight).  No COI  No industrial sponsorship. |
|  | Low | Unclear | Unclear | Unclear | High | High | High |
| Venn, New Zealand  (Venn et al., 2010) | No description | No description | No description of blinding of participants and dieticians | No description | Intention to treat analysis was employed. Missing values were imputed using chained equations. Dropout rate was 44% is one group and 11% in the other | No published protocol | No imbalance in baseline characteristics  No COI  No industrial sponsorship. |
|  | Unclear | Unclear | Unclear | Unclear | High | Unclear | Low |
| Foster-Schubert, USA  (Foster‐Schubert et al., 2012) | “*The random assignment was generated by a computerized program, stratified according to BMI… and participants’ self-reported race/ethnicity…”* | No description | No description of blinding of participants and exercise physiologists.  Blinded trained study personnel  Dietitians were not blinded as they delivered the intervention. | “*All study measurements were obtained by trained study personnel who were blinded to participants’ randomization status*” | Dropout rate was between 8 and 12%, with no differences between groups. | No published protocol | Imbalance in baseline characteristics (ethnicity and fat intake).  No COI  No industrial sponsorship. |
|  | Low | Unclear | Unclear | Low | Low | Unclear | High |
| Fernandez,  Spain  (Fernández et al., 2012) | *“Random allocation to each dietary group was performed by the main investigator (MDBP) by using the Web site Randonuzation.com (http://www.randomization.com) upon the request of the study dietitian (ACF) after the eligibility of a participant was confirmed and assignment to either of the insulin resistance or insulin sensitivity groups had been performed”* | Allocation was done by the main investigator | No description of participants’ blinding.  Dietician not blinded | *“The medical assessments were blinded to the type of diet being followed*” | 40-60% dropout | No published protocol | Waist Circumference differed across groups  Funding and COI: No description |
|  | Low | High | Unclear | Low | High | Unclear | High |
| Wycherley, Australia  (Wycherley et al., 2012) | *“Participants were blocked, matched for age and body mass index, then randomized by the trial coordinators using computer-generated random number allocation”* | No description | No description of blinding of dietitians and nurses. | No details regarding the blinding of outcome assessors, specialist for body composition scanning | 36-40% dropout at 1 year and no description of imputation methods | ACTRN12606000002583 registered protocol. Pre-specified outcomes | Significant difference in Triglyceride level at baseline No COI  No industrial funding |
|  | Low | Unclear | Unclear | Unclear | High | Low | Low |
| Griffin, Australia  (Griffin et al., 2013) | Randomized table from statistic book | *Random allocation (sealed opaque envelopes),* information taken from the online protocol | Participants were blinded.  “*Participants were blind to the macronutrient composition and were informed eating plans differed only in the type of meat (i.e. red or white)”.*  No description of dietitians blinding  No other personnel involved in the implementation of the intervention. | Single blinded (participants); outcome assessors not blinded. | 49% dropout among all participants | ACTRN12609000307202 and pre-specified outcomes | No imbalance in baseline characteristics  No COI  No industrial sponsorship |
|  | Low | Low | Low | High | High | Low | Low |
| Jesudason, USA  (Jesudason et al., 2013) | “*Participants were block randomly assigned by trial coordinators to one of 2 diets by using the Clinstat program (Martin Bland; available from:*[*http://www-users.york.ac.uk/∼mb55/soft/soft.htm*](http://www-users.york.ac.uk/~mb55/soft/soft.htm)*) matched for age, weight, smoking status, and use of selective serotonin reuptake inhibitor drugs*” | No description | Participants and dieticians were not blinded.  “*Although the investigators were blinded until the end of the study, subjects and dietitians were unblinded*”  Investigators were blinded | Outcome assessor not described.  Investigators were blinded, but not sure whether they were involved in the measurement of participants anthropometrics | >20% dropout at 1 year | Registered protocol: ACTRN12608000229370 and pre-specified outcomes | No imbalance in baseline characteristics  No COI  No description of funding. |
|  | Low | Unclear | Unclear | Unclear | High | Low | Low |
| Rusu, Romania (Rusu et al., 2013) | *“Independently done computer randomization was used to allocate numbers and divide the patients into two groups. Randomization was done by block design to ensure equal numbers in each group for every 4 subjects recruited”* | No description | No description of blinding of participants and personnel | Outcome assessors not mentioned. | No mention of missing data; Dropout rate is 15% is one group and 3% in the other | No published protocol | No imbalance in baseline characteristics  No COI  No industrial sponsorship |
|  | Low | Unclear | Unclear | Unclear | High | Unclear | Low |
| Bazzano, USA (Bazzano et al., 2014) | “*We used a computer-generated randomization, stratified by sex, to allocate participants to 1 of the 2 diet groups*” | No description | No description of blinding of participants  Personnel other that dieticians were present such as staff that gave instructions, certified staff making recalls | No description | 17% dropout per arm, reasons described and evenly distributed | Registered protocol: NCT00609271. Outcomes pre-specified | No imbalance in baseline characteristics  No COI  No industrial sponsorship |
|  | Low | Unclear | Unclear | Unclear | Low | Low | Low |
| Mellberg, Sweden  (Mellberg et al., 2014) | *“Block randomization with a block size of four and an allocation ratio of 1:1 was done by a statistician blinded to the study”.*  No description of the method for randomization. | No description | No description of blinding of participants.  Dieticians not blinded  Personnel other than dieticians were present. They were all blinded. *“All study personnel (except the dieticians were blinded to the dietary allocation of the participants)* | All study personnel were blinded, except dieticians. It is not clear whether study personnel include outcome assessors | 23-37% dropout per arm; no description of imputation methods | NCT00692536 registered protocol; pre-defined outcomes | No imbalance in baseline characteristics  No COI  No industrial sponsorship |
|  | Unclear | Unclear | Unclear | Unclear | High | Low | Low |
| Ma, USA  (Ma et al., 2015) | *“Within each strata, participants were randomized to the two interventions in randomly permuted blocks of size 6 via the RALLOC procedure using Stata to ensure that the distributions of gender and BMI were similar between the two interventions”* | No description | No description of participants and dieticians blinding | “Research team staff evaluating outcomes and dieticians performing the 24h recall were blinded to intervention assignment” | Similar rates of drop out across arms (18-19%), reasons described and evenly distributed across arms. and used a reliable imputation method | [NCT00911885](https://clinicaltrials.gov/ct2/show/NCT00911885) registered protocol.  and pre-defined outcomes | No meaningful differences were found between the groups for baseline characteristic.  No COI  No industrial sponsorship |
|  | Low | Unclear | Unclear | Low | Low | Low | Unclear |
| Santanasto, USA  (Santanasto et al., 2015) | No description | No description | No description of participants and dieticians blinding | No description | Dropout rate in the overall population 11%. | Registered protocol: NCT00714506. Weight mentioned in the pre-specified outcomes | No imbalance in baseline characteristics.  No COI  No industrial sponsorship |
|  | Unclear | Unclear | Unclear | Unclear | Low | Low | Low |
| Brinkworth, Australia (Brinkworth et al., 2016) | “Randomized” without further details | No description | No description of participants and dieticians blinding | No description | Dropout rate of 26%. No mention of imputation method of missing data | No published protocol | No imbalance in baseline characteristics  No COI  No industrial sponsorship |
|  | Unclear | Unclear | Unclear | Unclear | High | Unclear | Low |
| Gepner, Israel  (Gepner et al., 2017) | “*The randomization was performed with an allocation ratio of 1:1 to the 2 treatment groups, within strata of baseline VAT area for the first randomization, and of address of residence …. in the second randomization (in blocks of 2*)”  No additional details on methodology of sequence generation. | No description | Participants not blinded  *“The participants were aware of their assigned intervention (open label)”*  Blinding of dieticians not described.  Study personnel, fitness instructors and technicians, were blinded. | *“Study investigators assessing outcomes were blinded to the group assignments”* | Dropout 12-15% and reasons for dropout described and evenly distributed across arms | Registered protocol: NCT01530724; Outcomes pre-specified | No imbalance in baseline characteristics  No COI  No industrial sponsorship |
|  | Unclear | Unclear | Low | Low | Low | Low | Low |
| Gardner, USA  (Gardner et al., 2018) | *“Allocation sequence determined by computerized random-number generation (Blockrand in R version 3.4.0; R Project for Statistical Computing) in block sizes of 8 (with 4 individuals going to each diet) by a statistician not involved in intervention delivery or data collection”* | No description | No description of participants blinding. *“Participants did not learn of their diet group assignment until they completed all baseline measures and attended their first intervention class”*  Dieticians not blinded  No other personnel involved in the implementation of the intervention | *“Staff who measured outcomes were blinded to diet assignment, genotype pattern, INS-30, and diet assignment”*  Dieticians were blinded to laboratory measurements and genotypes, but not to anthropometrics | Dropout 21-22% per arm, similar reasons and imputation methods described | NCT01826591 registered protocol and outcomes pre-specified | No imbalance in baseline characteristics  No COI  No industrial sponsorship |
|  | Low | Unclear | Unclear | Low | High | Low | Low |
| Pavic, Croatia  (Pavić et al., 2019) | “*Randomization was done by flipping a coin*”, but this is not an appropriate method for sample size <100 participants | No description | Since it is a single blinded study, participants did not know allocated diet.  Dieticians were not blinded | No description | Overall dropout rate was high: 33%. | Registered protocol: NCT02259244; outcomes pre-specified | No imbalance in baseline characteristics except for the number of patients on antihypertensive medications. No COI  No mention of name of funding agency. |
|  | High | Unclear | Low | Unclear | High | Low | Unclear |

COI: Conflict of Interest, USA: United States of America

**Table S4: Summary of sub-group analyses for the comparison High Fat/Low Carbohydrate versus Moderate Macronutrients diets**

|  | **Intervention duration**  **(12 months**  **Versus**  **> 12 months)** | **Co-intervention** | **Patients’ age**  **Age < 50 years**  **Versus**  **Age ≥ 50 years** | **Gender**  **Men >75% of the population**  **versus**  **Women >75% of the population** |
| --- | --- | --- | --- | --- |
| Change in weight (kg) | 12 months  MD -0.93(-1.73; -0.14)  >12 months  MD -1.56(-2.69; -0.43)  p-subgroup 0.38 | Physical activity only  MD 0.53(-1.21; 2.28)  Behavioral therapy only  MD -1.97(-4.61; 0.67)  Both  MD -1.19(-2.25; -0.12)  None  MD -1.17(-2.02; -0.33)  p-subgroup 0.29 | Age < 50 years  MD -1.5(-2.45; 0.55)  Age ≥ 50 years  MD -0.59(-1.44; 0.40)  p-subgroup 0.15 | Men  MD -1.68(-2.86; -0.50)  Women  MD -1.39(-2.62; -0.16)  p-subgroup 0.74 |
| Change in BMI kg/m^2^ | 12 months  MD -0.19(-0.36; -0.02)  >12 months  MD -0.73(-1.23; -0.22)  p-subgroup 0.05 | Physical activity only  MD 0.23(-0.28; 0.73)  Behavioral therapy only  MD -0.7(-1.51; 0.11)  Both  MD -0.32(-0.59; -0.06)  None  MD -0.46(-0.78; -0.15)  p-subgroup 0.11 | Age < 50 years  MD -0.25 (-0.47; -0.02)  Age ≥ 50 years  MD -0.64(-1.21; -0.07)  p-subgroup 0.21 | - |
| Change in waist circumference (cm) | 12 months  MD -0.84(-2.05; 0.37)  >12 months  MD -0.70(-1.78; 0.37)  p-subgroup 0.87 | Physical activity only  MD 0.77(-1.24; 2.78)  Behavioral therapy only  MD 0.86(-0.72; 2.45)  Both  MD -1.82 (-3.65; 0.01)  None  MD -1.12(-2.11; -0.14)  p-subgroup 0.05 | Age < 50 years  MD -1.33(-2.49; -0.16)  Age ≥ 50 years  MD -0.06(-1.34; 1.22)  p-subgroup 0.15 | Men  MD -0.89(-1.98; 0.19)  Women  MD -0.71(-2.6; 1.17)  p-subgroup 0.87 |

MD: Mean Difference

**Figure S1: Risk of bias of studies assessing weight change**


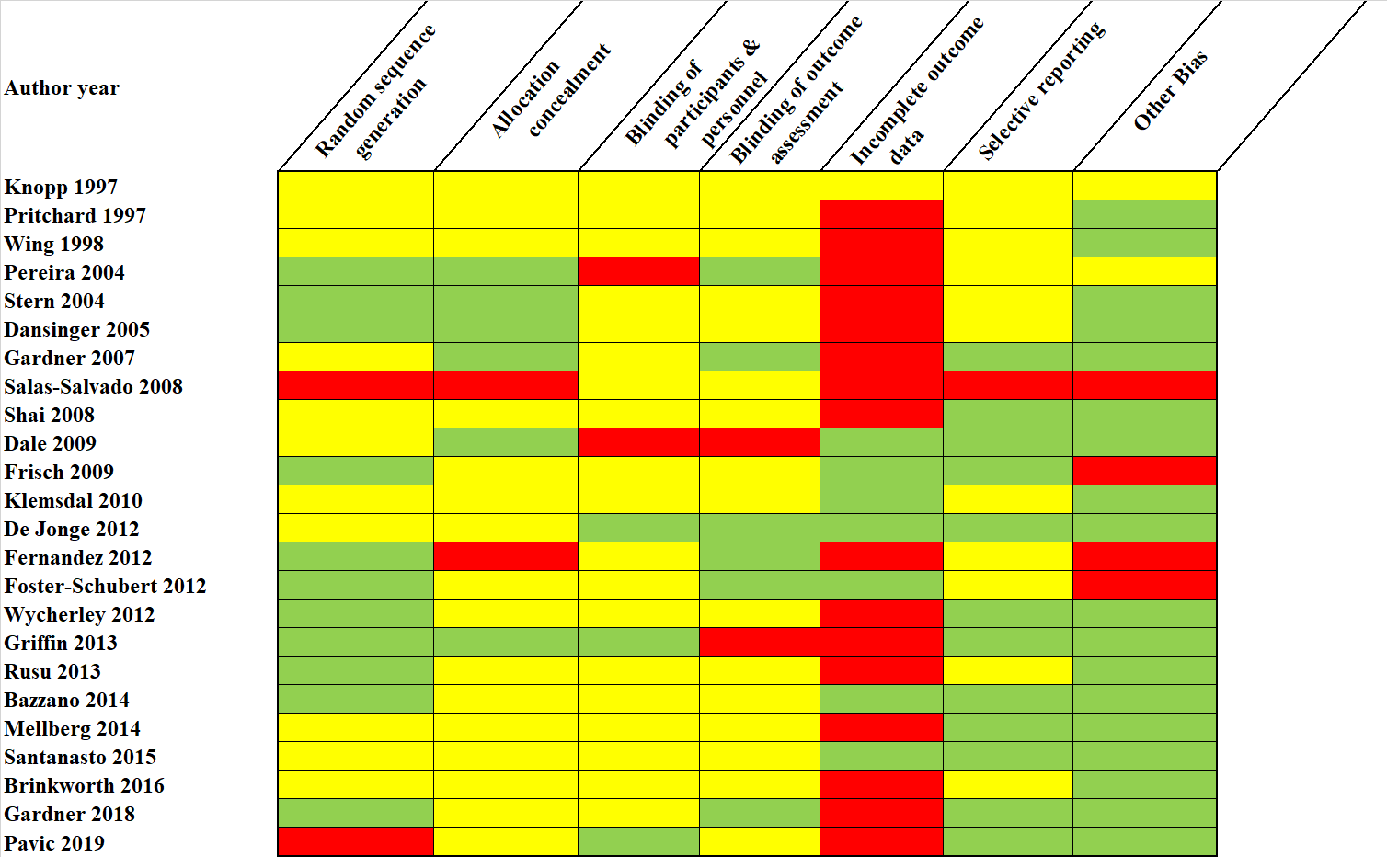


Green: Low risk of bias, Yellow: Unclear risk of bias, Red: High risk of bias.

**Figure S2: Risk of bias of studies assessing body mass index change**


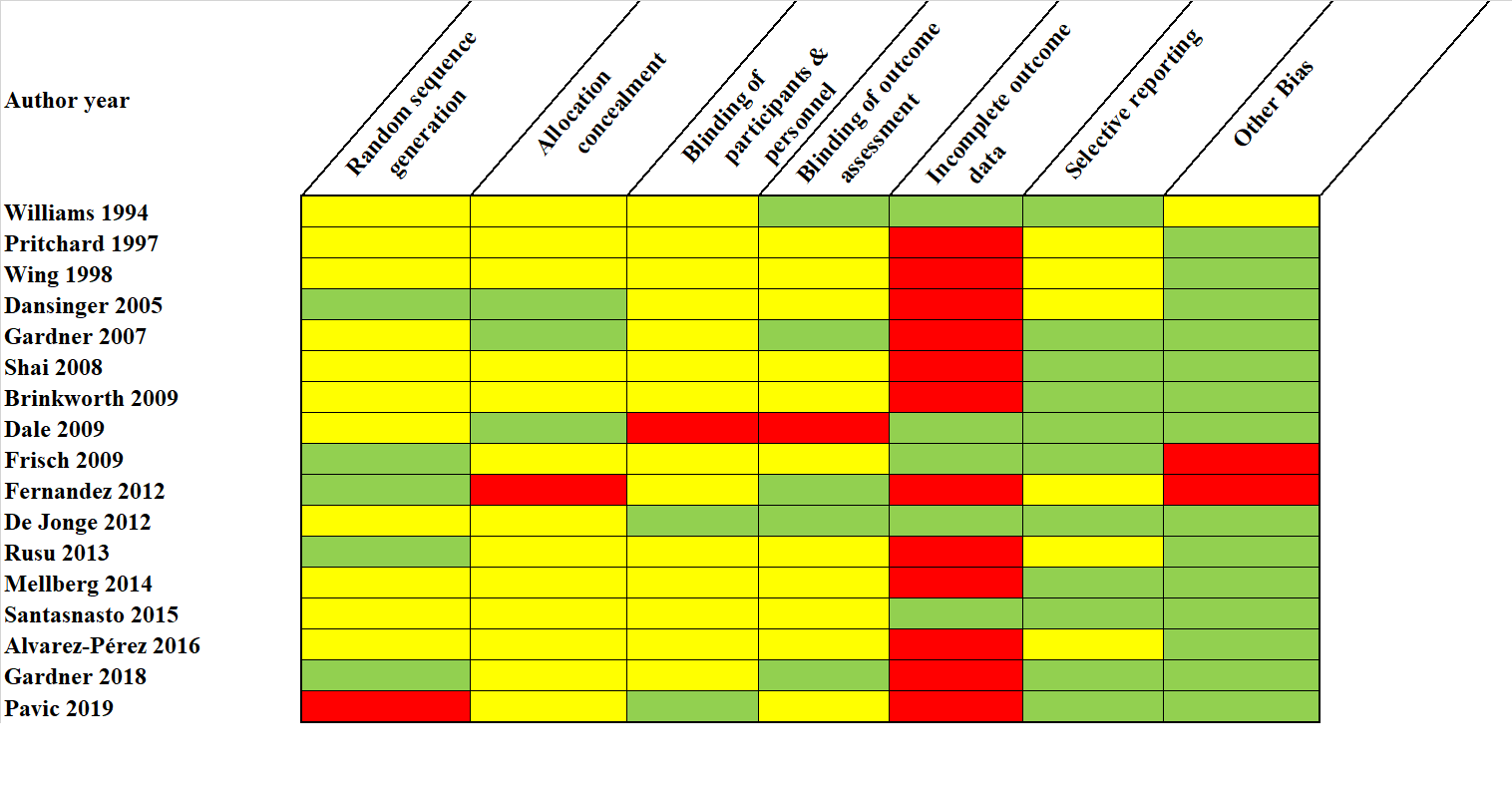


Green: Low risk of bias, Yellow: Unclear risk of bias, Red: High risk of bias.

**Figure S3: Risk of bias of studies assessing waist circumference change**


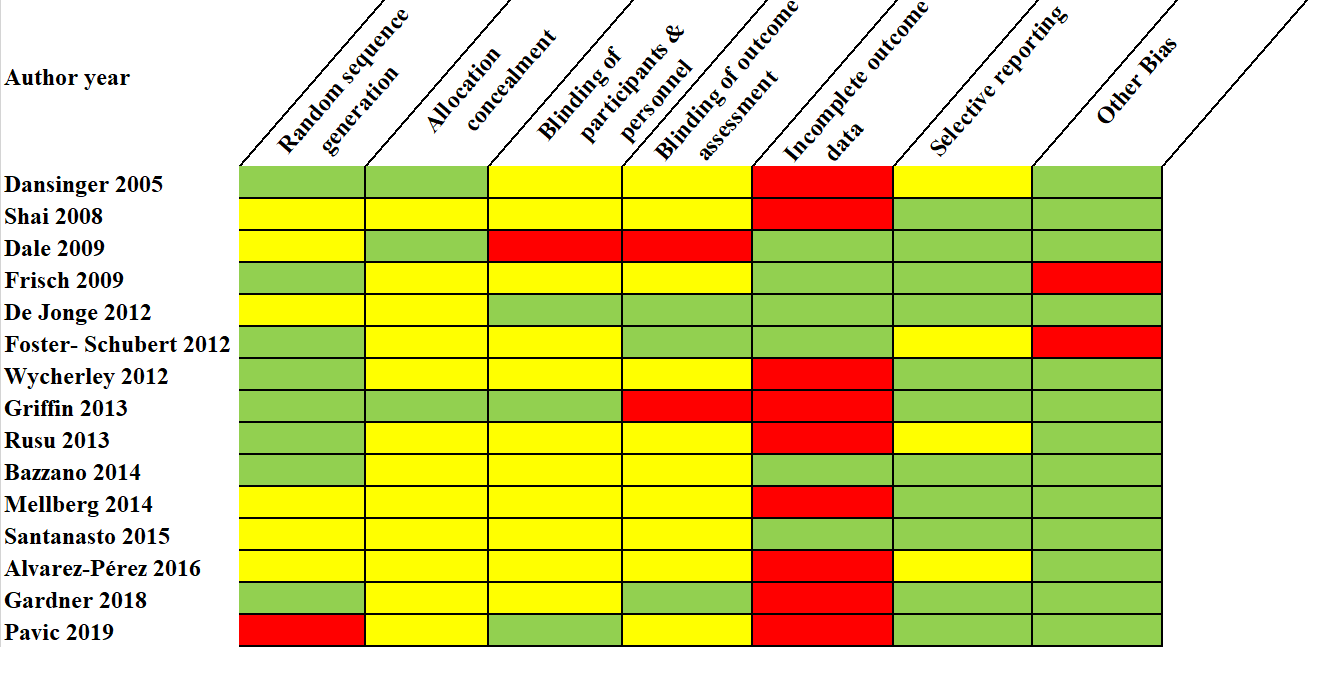


Green: Low risk of bias, Yellow: Unclear risk of bias, Red: High risk of bias.

**Figure S4: Funnel plot of High Fat Low Carbohydrate vs Moderate Macronutrient diets and weight change**


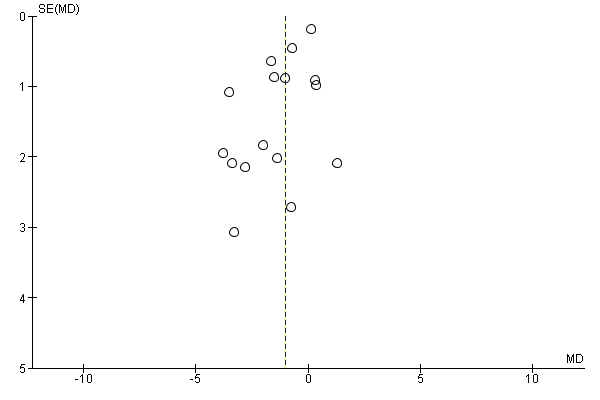


**Figure S5: Funnel plot of High Fat Low Carbohydrate vs Moderate Macronutrient diets and body mass index change**


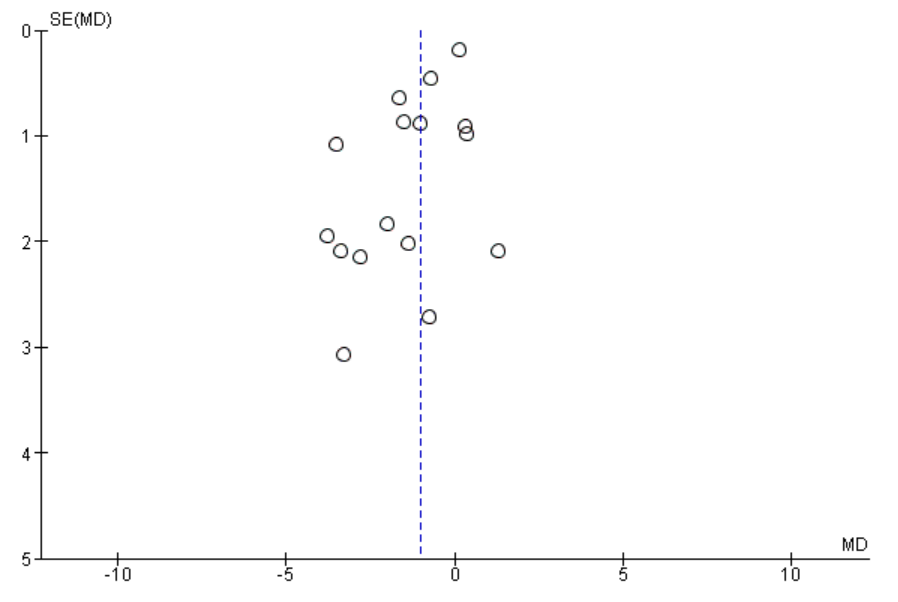


**Figure S6: Funnel plot of High Fat Low Carbohydrate vs Moderate Macronutrient diets and waist circumference change**


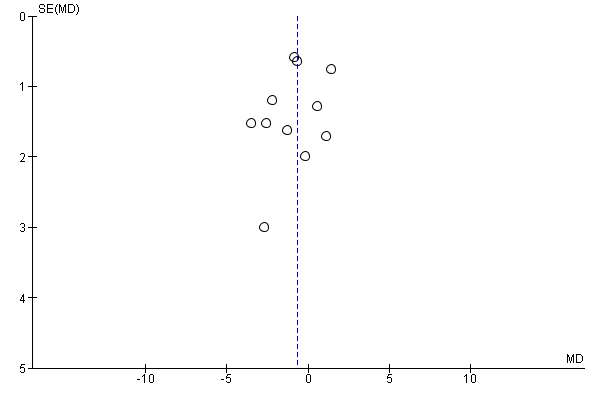


**References**

Acosta, A., Streett, S., Kroh, M.D., Cheskin, L.J., Saunders, K.H., Kurian, M., Schofield, M., Barlow, S.E., and Aronne, L. (2017). White paper AGA: POWER—practice guide on obesity and weight management, education, and resources. *Clinical gastroenterology and hepatology* 15**,** 631-649. e610.

Affairs, D.O.V. (2014). "VA/DoD clinical practice guideline for screening and management of overweight and obesity". Office of Quality and Performance publication Washington, DC).

Apovian, C.M., Aronne, L.J., Bessesen, D.H., Mcdonnell, M.E., Murad, M.H., Pagotto, U., Ryan, D.H., and Still, C.D. (2015). Pharmacological management of obesity: an Endocrine Society clinical practice guideline. *The Journal of Clinical Endocrinology & Metabolism* 100**,** 342-362.

Azadbakht, L., Mirmiran, P., Esmaillzadeh, A., and Azizi, F. (2007). Better dietary adherence and weight maintenance achieved by a long-term moderate-fat diet. *British journal of nutrition* 97**,** 399-404.

Bazzano, L.A., Hu, T., Reynolds, K., Yao, L., Bunol, C., Liu, Y., Chen, C.-S., Klag, M.J., Whelton, P.K., and He, J. (2014). Effects of low-carbohydrate and low-fat diets: a randomized trial. *Annals of internal medicine* 161**,** 309-318.

Brinkworth, G.D., Wycherley, T.P., Noakes, M., Buckley, J.D., and Clifton, P.M. (2016). Long-term effects of a very-low-carbohydrate weight-loss diet and an isocaloric low-fat diet on bone health in obese adults. *Nutrition* 32**,** 1033-1036.

Council, N.H.a.M.R. (2013). "The Clinical Practice Guidelines for the management of overweight and obesity in adults, adolescents and children in Australia is intended for use by clinicians including general practitioners. ", (ed.) A. Government. (Melbourne, Australia).

Dale, K.S., Mcauley, K.A., Taylor, R.W., Williams, S.M., Farmer, V.L., Hansen, P., Vorgers, S.M., Chisholm, A.W., and Mann, J.I. (2009). Determining optimal approaches for weight maintenance: a randomized controlled trial. *Cmaj* 180**,** E39-E46.

Dansinger, M.L., Gleason, J.A., Griffith, J.L., Selker, H.P., and Schaefer, E.J. (2005). Comparison of the Atkins, Ornish, Weight Watchers, and Zone diets for weight loss and heart disease risk reduction: a randomized trial. *Jama* 293**,** 43-53.

De Jonge, L., Bray, G.A., Smith, S.R., Ryan, D.H., De Souza, R.J., Loria, C.M., Champagne, C.M., Williamson, D.A., and Sacks, F.M. (2012). Effect of diet composition and weight loss on resting energy expenditure in the POUNDS LOST study. *Obesity* 20**,** 2384-2389.

Due, A., Toubro, S., Skov, A., and Astrup, A. (2004). Effect of normal-fat diets, either medium or high in protein, on body weight in overweight subjects: a randomised 1-year trial. *International journal of obesity* 28**,** 1283-1290.

Ebbeling, C.B., Leidig, M.M., Sinclair, K.B., Seger-Shippee, L.G., Feldman, H.A., and Ludwig, D.S. (2005). Effects of an ad libitum low-glycemic load diet on cardiovascular disease risk factors in obese young adults. *The American journal of clinical nutrition* 81**,** 976-982.

Excellence, N.I.F.H.a.C. (2014). "Obesity: identification, assessment and management".).

Fernández, A.C., Casariego, A.V., and Rodríguez, I.C. (2012). One-year effectiveness of two hypocaloric diets with different protein/carbohydrate ratios in weight loss and insulin resistance. *Nutricion hospitalaria* 27**,** 2093-2101.

Foster‐Schubert, K.E., Alfano, C.M., Duggan, C.R., Xiao, L., Campbell, K.L., Kong, A., Bain, C.E., Wang, C.Y., Blackburn, G.L., and Mctiernan, A. (2012). Effect of diet and exercise, alone or combined, on weight and body composition in overweight‐to‐obese postmenopausal women. *Obesity* 20**,** 1628-1638.

Frisch, S., Zittermann, A., Berthold, H.K., Götting, C., Kuhn, J., Kleesiek, K., Stehle, P., and Körtke, H. (2009). A randomized controlled trial on the efficacy of carbohydrate-reduced or fat-reduced diets in patients attending a telemedically guided weight loss program. *Cardiovascular diabetology* 8**,** 1-10.

Gardner, C.D., Kiazand, A., Alhassan, S., Kim, S., Stafford, R.S., Balise, R.R., Kraemer, H.C., and King, A.C. (2007). Comparison of the Atkins, Zone, Ornish, and LEARN diets for change in weight and related risk factors among overweight premenopausal women: the A TO Z Weight Loss Study: a randomized trial. *Jama* 297**,** 969-977.

Gardner, C.D., Trepanowski, J.F., Del Gobbo, L.C., Hauser, M.E., Rigdon, J., Ioannidis, J.P., Desai, M., and King, A.C. (2018). Effect of low-fat vs low-carbohydrate diet on 12-month weight loss in overweight adults and the association with genotype pattern or insulin secretion: the DIETFITS randomized clinical trial. *Jama* 319**,** 667-679.

Garvey, W.T., Mechanick, J.I., Brett, E.M., Garber, A.J., Hurley, D.L., Jastreboff, A.M., Nadolsky, K., Pessah-Pollack, R., and Plodkowski, R. (2016). American association of clinical endocrinologists and American college of endocrinology comprehensive clinical practice guidelines formedical care of patients with obesity. *Endocrine Practice* 22**,** 1-203.

Gepner, Y., Shelef, I., Schwarzfuchs, D., Cohen, N., Bril, N., Rein, M., Tsaban, G., Zelicha, H., Yaskolka Meir, A., and Tene, L. (2017). Intramyocellular triacylglycerol accumulation across weight loss strategies; Sub-study of the CENTRAL trial. *PloS one* 12**,** e0188431.

Griffin, H., Cheng, H., O'connor, H., Rooney, K., Petocz, P., and Steinbeck, K. (2013). Higher protein diet for weight management in young overweight women: a 12‐month randomized controlled trial. *Diabetes, Obesity and Metabolism* 15**,** 572-575.

Jensen, M.D., Ryan, D.H., Apovian, C.M., Ard, J.D., Comuzzie, A.G., Donato, K.A., Hu, F.B., Hubbard, V.S., Jakicic, J.M., Kushner, R.F., Loria, C.M., Millen, B.E., Nonas, C.A., Pi-Sunyer, F.X., Stevens, J., Stevens, V.J., Wadden, T.A., Wolfe, B.M., and Yanovski, S.Z. (2014). 2013 AHA/ACC/TOS Guideline for the Management of Overweight and Obesity in Adults. *Circulation* 129**,** S102-S138.

Jesudason, D., Nordin, B.C., Keogh, J., and Clifton, P. (2013). Comparison of 2 weight-loss diets of different protein content on bone health: a randomized trial. *The American journal of clinical nutrition* 98**,** 1343-1352.

Keogh, J.B., Brinkworth, G.D., and Clifton, P.M. (2007). Effects of weight loss on a low-carbohydrate diet on flow-mediated dilatation, adhesion molecules and adiponectin. *British Journal of Nutrition* 98**,** 852-859.

Klemsdal, T.O., Holme, I., Nerland, H., Pedersen, T.R., and Tonstad, S. (2010). Effects of a low glycemic load diet versus a low-fat diet in subjects with and without the metabolic syndrome. *Nutrition, Metabolism and Cardiovascular Diseases* 20**,** 195-201.

Knopp, R.H., Walden, C.E., Retzlaff, B.M., Mccann, B.S., Dowdy, A.A., Albers, J.J., Gey, G.O., and Cooper, M.N. (1997). Long-term cholesterol-lowering effects of 4 fat-restricted diets in hypercholesterolemic and combined hyperlipidemic men: the Dietary Alternatives Study. *Jama* 278**,** 1509-1515.

Ma, Y., Olendzki, B.C., Wang, J., Persuitte, G.M., Li, W., Fang, H., Merriam, P.A., Wedick, N.M., Ockene, I.S., and Culver, A.L. (2015). A randomized trial of single-versus multi-component dietary goals for metabolic syndrome. *Annals of internal medicine* 162**,** 248.

Marniemi, J., Seppänen, A., and Hakala, P. (1990). Long-term effects on lipid metabolism of weight reduction on lactovegetarian and mixed diet. *International journal of obesity* 14**,** 113-125.

Mellberg, C., Sandberg, S., Ryberg, M., Eriksson, M., Brage, S., Larsson, C., Olsson, T., and Lindahl, B. (2014). Long-term effects of a Palaeolithic-type diet in obese postmenopausal women: a 2-year randomized trial. *European journal of clinical nutrition* 68**,** 350-357.

Moyer, V.A. (2012). Screening for and management of obesity in adults: US Preventive Services Task Force recommendation statement. *Annals of internal medicine* 157**,** 373-378.

Pavić, E., Hadžiabdić, M.O., Mucalo, I., Martinis, I., Romić, Ž., Božikov, V., and Rahelić, D. (2019). Effect of the Mediterranean diet in combination with exercise on metabolic syndrome parameters: 1-year randomized controlled trial. *International journal for vitamin and nutrition research*.

Pereira, M.A., Swain, J., Goldfine, A.B., Rifai, N., and Ludwig, D.S. (2004). Effects of a low–glycemic load diet on resting energy expenditure and heart disease risk factors during weight loss. *Jama* 292**,** 2482-2490.

Physicians, A.a.O.F. (2013). "Diagnosis and management of obesity". (Leawood, KS, USA).

Pritchard, J.E., Nowson, C.A., and Wark, J.D. (1997). A worksite program for overweight middle-aged men achieves lesser weight loss with exercise than with dietary change. *Journal of the American Dietetic Association* 97**,** 37-42.

Rusu, E., Jinga, M., Enache, G., Rusu, F., Dragomir, A.D., Ancuta, I., Draguţ, R., Parpala, C., Nan, R., and Sima, I. (2013). Effects of lifestyle changes including specific dietary intervention and physical activity in the management of patients with chronic hepatitis C-a randomized trial. *Nutrition journal* 12**,** 1-12.

Sacks, F.M., Bray, G.A., Carey, V.J., Smith, S.R., Ryan, D.H., Anton, S.D., Mcmanus, K., Champagne, C.M., Bishop, L.M., and Laranjo, N. (2009). Comparison of weight-loss diets with different compositions of fat, protein, and carbohydrates. *New England Journal of Medicine* 360**,** 859-873.

Salas-Salvadó, J., Fernández-Ballart, J., Ros, E., Martinez-Gonzalez, M.-A., Fitó, M., Estruch, R., Corella, D., Fiol, M., Gómez-Gracia, E., and Arós, F. (2008). Effect of a Mediterranean diet supplemented with nuts on metabolic syndrome status: one-year results of the PREDIMED randomized trial. *Archives of internal medicine* 168**,** 2449-2458.

Santanasto, A., Newman, A., Strotmeyer, E., Boudreau, R., Goodpaster, B., and Glynn, N.W. (2015). Effects of changes in regional body composition on physical function in older adults: a pilot randomized controlled trial. *The journal of nutrition, health & aging* 19**,** 913-921.

Schutz, D.D., Busetto, L., Dicker, D., Farpour-Lambert, N., Pryke, R., Toplak, H., Widmer, D., Yumuk, V., and Schutz, Y. (2019). European practical and patient-centred guidelines for adult obesity management in primary care. *Obesity facts* 12**,** 40-66.

Shai, I., Schwarzfuchs, D., Henkin, Y., Shahar, D.R., Witkow, S., Greenberg, I., Golan, R., Fraser, D., Bolotin, A., and Vardi, H. (2008). Weight loss with a low-carbohydrate, Mediterranean, or low-fat diet. *New England Journal of Medicine* 359**,** 229-241.

Stern, L., Iqbal, N., Seshadri, P., Chicano, K.L., Daily, D.A., Mcgrory, J., Williams, M., Gracely, E.J., and Samaha, F.F. (2004). The effects of low-carbohydrate versus conventional weight loss diets in severely obese adults: one-year follow-up of a randomized trial. *Annals of internal medicine* 140**,** 778-785.

Sukumar, D., Ambia‐Sobhan, H., Zurfluh, R., Schlussel, Y., Stahl, T.J., Gordon, C.L., and Shapses, S.A. (2011). Areal and volumetric bone mineral density and geometry at two levels of protein intake during caloric restriction: a randomized, controlled trial. *Journal of bone and mineral research* 26**,** 1339-1348.

Tanumihardjo, S.A., Valentine, A.R., Zhang, Z., Whigham, L.D., Lai, H.J., and Atkinson, R.L. (2009). Strategies to increase vegetable or reduce energy and fat intake induce weight loss in adults. *Experimental Biology and Medicine* 234**,** 542-552.

Venn, B.J., Perry, T., Green, T.J., Skeaff, C.M., Aitken, W., Moore, N.J., Mann, J.I., Wallace, A.J., Monro, J., and Bradshaw, A. (2010). The effect of increasing consumption of pulses and wholegrains in obese people: a randomized controlled trial. *Journal of the American College of Nutrition* 29**,** 365-372.

Wharton, S., Lau, D.C., Vallis, M., Sharma, A.M., Biertho, L., Campbell-Scherer, D., Adamo, K., Alberga, A., Bell, R., and Boulé, N. (2020). Obesity in adults: a clinical practice guideline. *Cmaj* 192**,** E875-E891.

Williams, P.T., Krauss, R.M., Stefanick, M.L., Vranizan, K.M., and Wood, P.D. (1994). Effects of low-fat diet, calorie restriction, and running on lipoprotein subfraction concentrations in moderately overweight men. *Metabolism* 43**,** 655-663.

Wing, R.R., Venditti, E., Jakicic, J.M., Polley, B.A., and Lang, W. (1998). Lifestyle intervention in overweight individuals with a family history of diabetes. *Diabetes care* 21**,** 350-359.

Wycherley, T., Brinkworth, G., Clifton, P., and Noakes, M. (2012). Comparison of the effects of 52 weeks weight loss with either a high-protein or high-carbohydrate diet on body composition and cardiometabolic risk factors in overweight and obese males. *Nutrition & diabetes* 2**,** e40-e40.
